# Data Imputation and Body Weight Variability Calculation Using Linear and Nonlinear Methods in Data Collected From Digital Smart Scales: Simulation and Validation Study

**DOI:** 10.2196/17977

**Published:** 2020-09-11

**Authors:** Jake Turicchi, Ruairi O'Driscoll, Graham Finlayson, Cristiana Duarte, A L Palmeira, Sofus C Larsen, Berit L Heitmann, R James Stubbs

**Affiliations:** 1 School of Psychology The University of Leeds Leeds United Kingdom; 2 Faculdade de Motricidade Humana Universidade de Lisboa Lisbon Portugal; 3 Research Unit for Dietary Studies The Parker Institute Bispebjerg and Frederiksberg Hospital The Capital Region Copenhagen Denmark; 4 The Boden Institute of Obesity Nutrition and Eating disorder University of Sydney Sydney Australia; 5 Department of Public Health Section for General Medicine University of Copenhagen Copenhagen Denmark

**Keywords:** weight variability, weight fluctuation, weight cycling, weight instability, imputation, validation, digital tracking, smart scales, body weight, energy balance

## Abstract

**Background:**

Body weight variability (BWV) is common in the general population and may act as a risk factor for obesity or diseases. The correct identification of these patterns may have prognostic or predictive value in clinical and research settings. With advancements in technology allowing for the frequent collection of body weight data from electronic smart scales, new opportunities to analyze and identify patterns in body weight data are available.

**Objective:**

This study aims to compare multiple methods of data imputation and BWV calculation using linear and nonlinear approaches

**Methods:**

In total, 50 participants from an ongoing weight loss maintenance study (the NoHoW study) were selected to develop the procedure. We addressed the following aspects of data analysis: cleaning, imputation, detrending, and calculation of total and local BWV. To test imputation, missing data were simulated at random and using real patterns of missingness. A total of 10 imputation strategies were tested. Next, BWV was calculated using linear and nonlinear approaches, and the effects of missing data and data imputation on these estimates were investigated.

**Results:**

Body weight imputation using structural modeling with Kalman smoothing or an exponentially weighted moving average provided the best agreement with observed values (root mean square error range 0.62%-0.64%). Imputation performance decreased with missingness and was similar between random and nonrandom simulations. Errors in BWV estimations from missing simulated data sets were low (2%-7% with 80% missing data or a mean of 67, SD 40.1 available body weights) compared with that of imputation strategies where errors were significantly greater, varying by imputation method.

**Conclusions:**

The decision to impute body weight data depends on the purpose of the analysis. Directions for the best performing imputation methods are provided. For the purpose of estimating BWV, data imputation should not be conducted. Linear and nonlinear methods of estimating BWV provide reasonably accurate estimates under high proportions (80%) of missing data.

## Introduction

### Background

Recently, the idea of remote health care monitored through a network of internet-connected devices, termed *The (Medical) Internet of Things* [1-3], has become popular, and in 2020, it is thought that 40% of internet of things–related technology is health related, accounting for US $117 billion [4]. With this information, precision medicine will become the future of health care. Frequently tracked body weight data are likely to become a valuable prognostic tool. We have already seen the incorporation of Wi-Fi–connected smart scales into research environments [5-7] accompanied by an increase in popularity and a decrease in costs among the general public. In weight management interventions, 80% and 60% of successful weight loss maintainers report self-weighing weekly and daily, respectively [8]. Regular self-weighing in research environments using tracking technologies will allow for more accurate recognition of body weight patterns, which are currently not well understood.

Body weight variability (BWV), that is, the variability around the overall trend in body weight, can be quantified from frequent body weight measures. Several recent studies have associated BWV with outcomes such as all-cause mortality [9-11], type 2 diabetes incidence [12], cardiovascular morbidity or mortality [13,14], and cancer [15]. Further indications suggest that BWV may serve as a potential prognostic tool for obesity [16,17] and as a risk factor in patients with heart failure [18]. However, significant heterogeneity exists in the methods used to process body weights and define BWV.

Although body weight is a reliable, valid, and simple metric to measure, its short-term dynamics are not well understood because, until recently, it has been difficult and time-consuming to make frequent longitudinal measures from an objective (ie, not self-reported) source, and previous studies estimating BWV generally use infrequent measurements (eg, every 6-12 months). Limitations in the methodologies used may contribute to the poor replicability of the results drawn from differing studies and populations: (1) definitions used and statistical inferences drawn from longitudinal weight data are extremely heterogeneous, (2) body weight changes are often measured retrospectively (by self-report) and/or infrequently (12 months apart), (3) overall trends in body weight (eg, weight increase or decrease) are often not addressed appropriately and may confound independent effects of BWV, and (4) missing data are often not appropriately addressed. Simple linear approaches to the measurement of BWV (such as root mean square error [RMSE] around the linear trend) are not able to fully differentiate the overall trend from the variability component. New strategies must be developed to improve the estimation of BWV.

Using frequent body weight measurements, few studies have examined weekly [19-21] or seasonal [21-23] patterns in body weight, although no study to our knowledge has estimated total BWV over the long term. In future, tracking technologies will become increasingly popular and accompanied by the acquisition of dense and complex data. Appropriate, validated, and accessible data processing methodologies must be devised to deal with such data. Such protocols have been developed for activity tracking [24-26], although they lack body weight tracking.

### Objectives

Recently, we collected body weight data from Wi-Fi–connected smart scales in individuals engaged in a weight loss maintenance trial (the NoHoW trial [27]) over 12 months. Therefore, we aimed to develop and evaluate a statistical protocol for analyzing frequent weight data by outlining an approach to cleaning, imputation, detrending, and estimating BWV using frequent body weight data to better inform future practices and quantify the magnitude of errors potentially associated with BWV estimates.

## Methods

### Materials and Subjects

For the purpose of this analysis, a subsample of 50 individuals were selected from the 1627 participants in the NoHoW trial. The NoHoW study is a 2×2 randomized controlled trial (RCT) testing the efficacy of an information and communications technology–based toolkit for delivering a weight loss maintenance intervention structured around evidence-based strategies related to self-regulation and emotion regulation in the United Kingdom (Leeds), Denmark (Copenhagen), and Portugal (Lisbon). Full inclusion and exclusion criteria and procedures can be found elsewhere [27]. Individuals who participated in the trial had reported ≥5% body weight loss in the 12 months before recruitment. The trial was registered with the ISRCTN registry (ISRCTN88405328). The study was conducted in accordance with the Helsinki Declaration. Ethical approval was granted by local institutional ethics committees at the University of Leeds (17-0082; February 27, 2017), the University of Lisbon (17/2016; February 20, 2017), and the Capital Region of Denmark (H-16030495; March 8, 2017).

The selection of the 50 participants for this analysis was based on those who had the greatest number of weight measurements in the first 12 months of the trial. Selecting those with the greatest completeness of data allowed for (1) better ability to simulate missingness and test imputation performance and (2) more valid baseline estimation of BWV, which can be used to test the agreement with other estimations (in comparison with missing simulated and imputed data). Although the study was an RCT, the structure of the RCT was not used, and all its arms were collapsed. Only 50 individuals were chosen to limit missingness in the observed data, which increases with sample size. All participants were provided with a Fitbit Aria (Fitbit Inc) body weight scale linked to a personalized Fitbit account, and the data were retrieved via the Fitbit app programming interface to a web-based data hub. The device has been shown previously by others to have excellent agreement with a calibrated research-grade SECA 769 scale [28]. Participants were instructed to weigh themselves at least twice per week for the duration of the trial. The characteristics of the participants are presented in Table 1.

**Table 1:** Participant characteristics (N=50).

Characteristics		Values	
**Gender, n (%)**			
	Male		15 (30)
	Female		35 (70)
Age (years), mean (SD)		49.2 (9.3)	
Weight (kg), mean (SD)		81.9 (15.4)	
BMI (kg/m^2^), mean (SD)		29.3 (6.8)	
Number of weight measurements, mean (SD)		336.0 (9.1)	

### Analysis Overview

**Figure 1:**
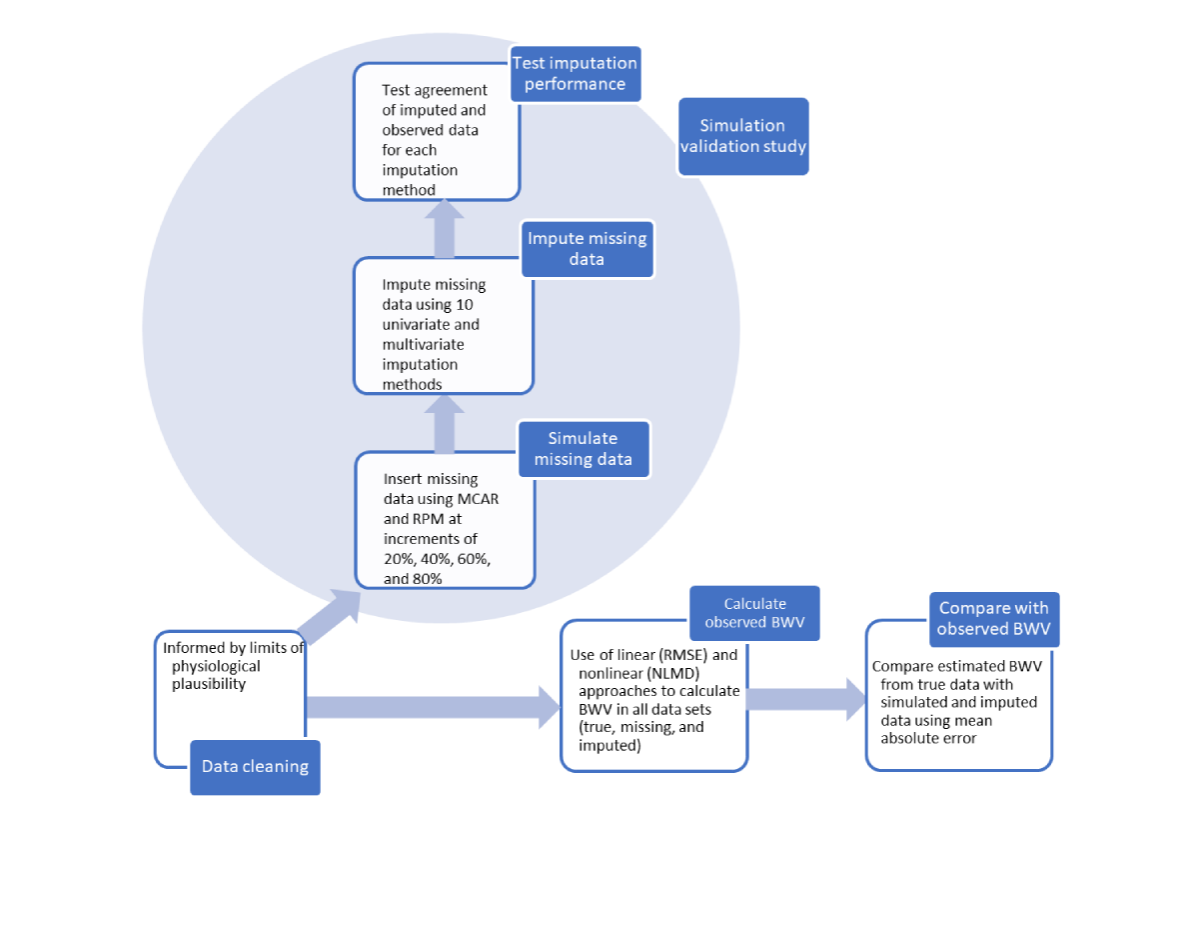
Study flow diagram. Outline of the study detailing the simulation validation study aimed to test imputation performance and calculation of linear and nonlinear body weight variability under conditions of true, missing, and imputed data sets with associated comparisons. BWV: body weight variability; MCAR: missing completely at random; NLMD: nonlinear mean deviation; RMSE: root mean square error; RPM: real patterns of missingness.

All statistical analyses were conducted using R version 3.5.1. All statistical codes used can be found in GitHub [29]. A flow diagram of the study is shown in Figure 1. First, we removed outliers based on the limits of physiological plausibility (detailed in Multimedia Appendix 1). Next, we used an amputation and imputation strategy outlined previously [30,31], which involved the simulation of missing data by 2 mechanisms: (1) removal completely at random and (2) removal informed by true patterns of missingness, followed by imputation using univariate and multivariate methods and performance testing using RMSE. Next, we calculated BWV in observed, simulated (ie, inserted missingness), and imputed data sets. This was done to test the accuracy of BWV estimation under conditions of incrementally missing data and when missing data were imputed. BWV was estimated using a commonly used linear approach (RMSE) and a nonlinear approach (nonlinear mean deviation, NLMD) devised for this analysis. Finally, we compared the agreement between BWV estimates from observed weight with those generated by simulated and imputed data sets under different conditions of missingness.

### Data Cleaning

Data outliers may be present for numerous reasons such as (1) decalibration of electronic scales, (2) inconsistent weighing conditions (eg, clothed vs unclothed or morning vs night), (3) weighing of another person of similar weight (which may register as a rapid weight change on the same Fitbit account), and (4) incorrect manual entry of body weight. We defined the limits of physiological plausibility for weight change over given periods, which can be seen in Multimedia Appendix 1. These limits were informed by substantial weight changes reported during rapid weight loss, such as those achieved by a very low–calorie diet [32,33], and rapid weight gain observed in intentional overfeeding studies [34,35]. It was deemed preferable to remove data based on these plausible limits than risk-removing potentially correct data.

### Data Removal

Typically, self-weighing is irregular, and thus, missing data are common. Missing data are generally categorized into missing at random (MAR), missing completely at random (MCAR), or not MAR [36]. Absence of body weight data may have identifiable mechanisms, for example, breaks in self-weighing may be indicative of weight gain [37]; however, these patterns may not be consistent between and within individuals. Data described as MCAR has no mechanism of missingness; however, data that are MAR are not related to the missing data but may be partially explained by the observed data. The data removal processes are described in detail in Multimedia Appendix 2. Briefly, to simulate missing data, we used 2 strategies. First, we inserted data using an MCAR strategy in increments of 20%, 40%, 60%, and 80%. For each of the 50 participants, we simulated 20 data sets per increment of missingness within each participant’s data, resulting in 4000 total MCAR-simulated data sets. One potential concern is that missing data in observed data are not entirely MCAR; therefore, MCAR simulation may not be representative of true missingness. To address this, we selected 20 random participants (for each increment of missingness) from our entire NoHoW study sample of 1627 individuals with approximately 20%, 40%, 60%, and 80% missing data and imposed these missing patterns on our 50-participant sample (with a near-complete data), resulting in 4000 simulated data sets with real patterns of missingness (RPM) data. Removing 20%, 40%, 60%, and 80% data left a mean of 255 (SD 54.5), 209 (SD 36.2), 144.8 (SD 50.1), and 67 (SD 40.1) available data points within a year (bearing in mind some data was missing in the original samples).

### Data Imputation

Data imputation can be broadly divided into univariate and multivariate approaches. Univariate methods impute missing data based on information gained from a single variable (in this case, a time series [TS] of body weights), whereas multivariate algorithms can be used to infer predictive value from related variables [38] through regression, clustering, or even advanced deep learning techniques. In a remote health care setting, many potentially useful variables for imputing weight data may not be collected (eg, information on psychology and behavior or physiological features), in which case univariate imputation may be necessary. The imputation of univariate TS data lends itself to a limited number of techniques that have been reviewed previously [30].

In total, 7 univariate imputation algorithms and 3 multivariate analyses were run on all missingness-simulated data sets. Univariate methods included (1) linear interpolation; (2) cubic spline interpolation; (3) Stine interpolation; (4) exponentially weighted moving average (EWMA); (5) structural modeling with Kalman smoothing (SMKS); (6) AutoRegressive Integrated Moving Average (ARIMA) state-space representation and Kalman smoothing (ASSRKS), all from the impute TS package [39]; and finally (7) an approach using the Friedman super smoother on nonseasonal data or seasonal decomposition on seasonal data followed by interpolation (TsClean) from the forecast package [40]. Each method is described briefly in Textbox 1. Illustrated examples using a single participant, showing imputation of 40% and 80% missingness by each imputation method, are provided in Multimedia Appendix 3. Multivariate imputation techniques, namely, 2 machine learning techniques (a K-nearest neighbors [KNN] method from the Data Mining with R (DMwR) package [41] and a random forest [RF] method from the MissForest package [42]) and a regression-based technique using predictive means matching (PMM) from the multivariate imputation by the chained equations (MICE) package [43] are described in Textbox 1. To maximize the usability of these methods where further information on participants were not available, we used only the day number and the day of the week as predictive variables for multivariate imputation.

**Description of univariate time series imputation methods used.**

Linear interpolation

- This method looks for a straight line that passes between 2 values (Xa and Xb), where the imputed values are bound between Xa and Xb. It has been demonstrated to be efficient when predicting values with constant rate of change [44], however, it tends to smooth data rather than impute variability

Spline interpolation

- This method fits local polynomial functions, which are connected at each end to form a spline, creating a succession of cubic splines over successive intervals of the data [45]. The order of the polynomial can be defined manually. The approach benefits from its nonlinear approach; however, its ability to predict oscillations from univariate data is limited [46]

Stine interpolation

- This is an advanced interpolation method where interpolation occurs based on (1) whether values of the ordinates of the specified points change monotonically and (2) the slopes of the line segments joining the specified points change monotonically. It produces a smoothed imputation known to be robust against sporadic outliers and performs better than spline interpolations, where abrupt changes are observed [47]

Exponentially weighted moving average (EWMA)

- This approach calculates the EWMA by assigning the value of the moving average window, which is user defined; the mean, thereafter, is calculated from equal number of observations on either side of a central missing value. The weighting factors decrease exponentially the greater distance from the missing value

Structural modeling with Kalman smoothing

- This method aims to identify the structure (trend, seasonality, and error) in a time series (TS). Unlike AutoRegressive Integrated Moving Average (ARIMA) state-space approaches where each component is eliminated, these components are used to inform imputation of missing data. Kalman filter and smoothing works in 2 steps to (1) produce estimates of the current state variables, along with their uncertainties, and (2) update estimates using a moving average to give a smoothing effect [48]. The Kalman smoother is given the entire sample and is not locally weighted. The Kalman smoother is robust to disparate observation periods (eg, when observations are made weekly and monthly in one TS) [49]

ARIMA state-space representation and Kalman smoothing

- This method converts the TS to an ARIMA model by decomposing the trend, seasonality, and error through a differencing protocol, resulting in a stationary TS where means and covariances would remain invariant over time [31]. Next, a Kalman smoother is applied as above

TsClean [40]

- This method first assesses evidence of seasonality. If present, a robust seasonal-trend decomposition for seasonal series is conducted followed by linear interpolation. If no seasonality is present, Friedman’s super smoother [50] is applied followed by linear interpolation

K-nearest neighbors [41]

- For every observation to be imputed, this algorithm locates *k* closest observations based on the Euclidean distance [51] and computes the weighted average (weighted based on distance) of these *k* observations

Random forest [42]

- This method is an extension of typical classification and regression, which generates predictive models that recursively subdivide the data based on values of the predictor variables. It does not rely on parametric assumptions and can accommodate nonlinear interactions, although it may be prone to overfitting [51]

Predictive means matching [43]

- For each missing entry, this method generates a small set of candidate donors from all complete cases that have predicted values closest to the predicted value for the missing entry. One donor is randomly drawn from the candidates, and the observed value of the donor is taken to replace the missing value. The assumption is the distribution of the missing cell is the same as the observed data of the candidate donors

### Estimating Body Weight Variability

**Figure 2:**
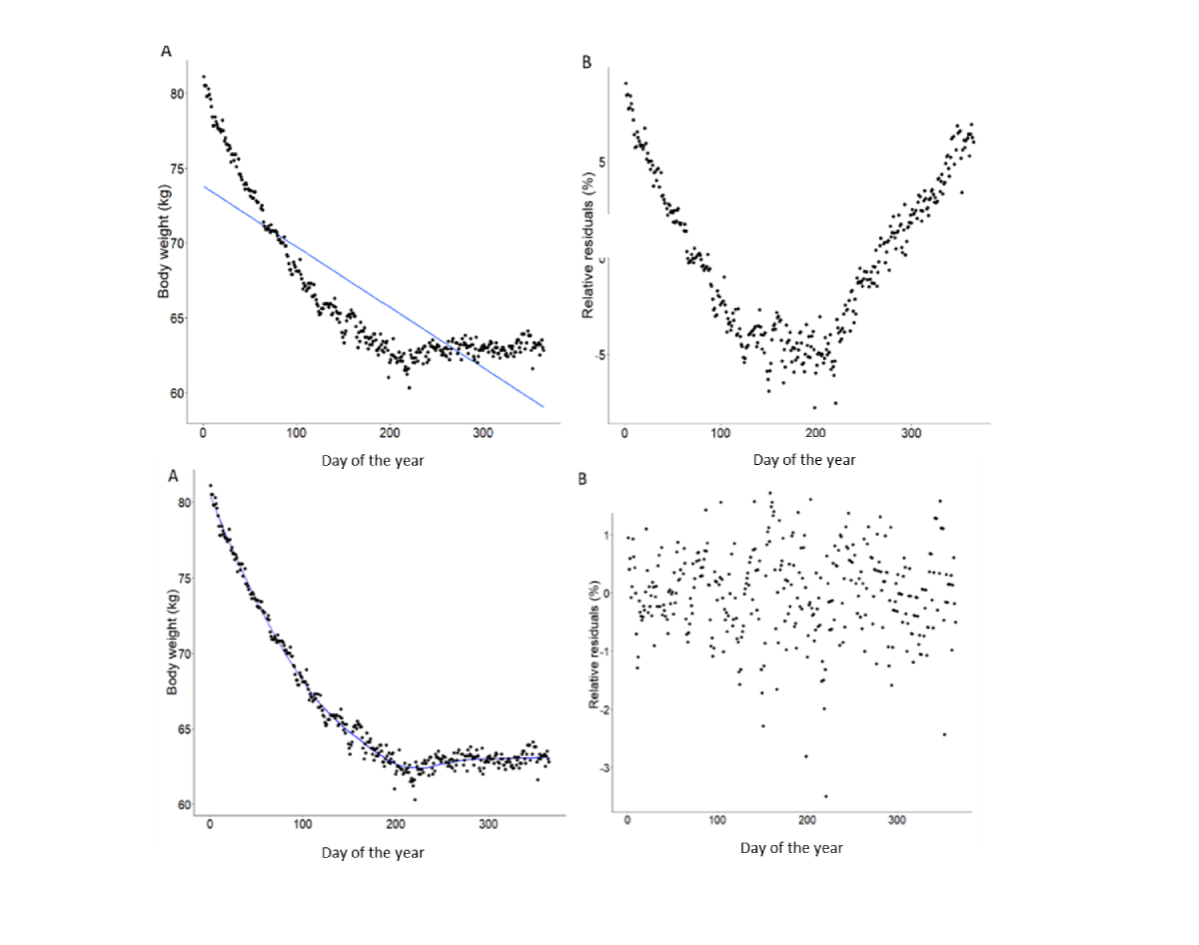
Performance summaries of univariate and multivariate imputation. Boxplots of the errors associated with imputation of body weight data collected by smart scales. Data was removed by a missing completely at random algorithm (left plots) and also informed by real patterns of missingness (right plots) in increments of 20%, 40%, 60% and 80%. Imputation was done by 7 univariate methods (top plots) and 3 multivariate methods (bottom plots). Root mean square error was used as the performance metric. ASSRKS: ARIMA state-space representation and Kalman smoothing; EWMA: exponentially weighted moving average; KNN: K-Nearest neighbors; Lin Int: linear interpolation; PMM: predictive means matching; RF: random forest; RMSE: root mean square error; SMKS: structural modelling with Kalman smoothing; Spline int: spline interpolation; Stine int: stine interpolation.

We estimated BWV using 2 discrete methods in the observed data as well as in all simulated and imputed data sets. These methods are illustrated in Figure 2 for linear (top) and nonlinear (bottom) approaches. First, the RMSE method was used by calculating the relative residual error of the linear relationship between body weight and time (Figure 2). Relative residuals were produced by dividing the centered weight by the observed weight at each time point (Figure 2). This method is commonly used to assess BWV in epidemiological research [16,52-56], although it is limited by the assumption of linearity of body weight change. For example, if an individual displays a curvilinear weight trajectory (such as in Figure 2), then the residuals from a linear trendline will be substantially different from those from a nonlinear trendline. To overcome this, we devised a nonlinear approach detailed below.

First, the series of body weights was detrended for each individual. Detrending is a necessary step in the decomposition of a TS. It can be used to isolate the variability component of the series from the overall trend, resulting in a combination of seasonal patterns (eg, any repetitive patterns including within-week) and random noise. First, a locally estimated scatterplot smoothing (LOESS) regression was fitted to each participant (Figure 2). LOESS regression is a nonlinear, nonparametric smoothing tool. Owing to its nonparametric approach, it does not assume previous specifications about the structure of the data, thus allowing for visual representation of relationships that do not conform to any structure [57]. LOESS regressions were conducted with the *stats* package in R [58]. It employs quadratic polynomial models on a moving collection of data points (termed a *neighborhood*) in a TS [59]. The size of the neighborhood is user defined and referred to as the *span* of the LOESS model, with greater spans creating more smooth trends because of using a wider collection of surrounding data points, whereas shorter spans resulting in closer fitting to the data. The span fits data based on the number of available data; therefore, when fitting the LOESS to data with missingness, the span must be reactive to the number of weight measurements available. To address this, we generated a linear relationship between the span and the number of available data, which resulted in a similar BWV estimation under varying conditions of missingness. Finally, a polynomial order of 2 was used in the model based on the nonlinearity of body weight data, as suggested previously [57].

The detrending process centers body weight around 0. The centered weights were converted to relative centered weights by dividing the centered weight by the observed weight at each time point (Figure 2). This gives an estimate of the relative deviation from the nonlinear trend. BWV was estimated by taking the mean of the centered relative residuals (which act as a proxy of the mean relative deviation from the trend on each day).

### Data Availability

There are legal restrictions on sharing data from this study that contain potentially identifying or sensitive personal information. The restrictions are imposed by the Danish Data Protection Agency Data used in this study will be made available upon request after application to the NoHoW data controller (the James Hutton Institute). The application procedure can be obtained from the James Hutton Institute (DPO@hutton.ac.uk) or David Nutter (david.nutter@bioss.ac.uk).

## Results

### Imputation Performance

All imputation algorithms were run on each simulated data set, generating 28,000 and 12,000 imputed data sets from MCAR and RPM simulations, respectively (4000 imputed data sets per imputation method). The performance of each imputed data set in comparison with the observed weight data was evaluated using the RMSE, which is commonly used for performance evaluation [60]. The RMSE was calculated using the following equation:

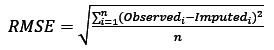



**Figure 3:**
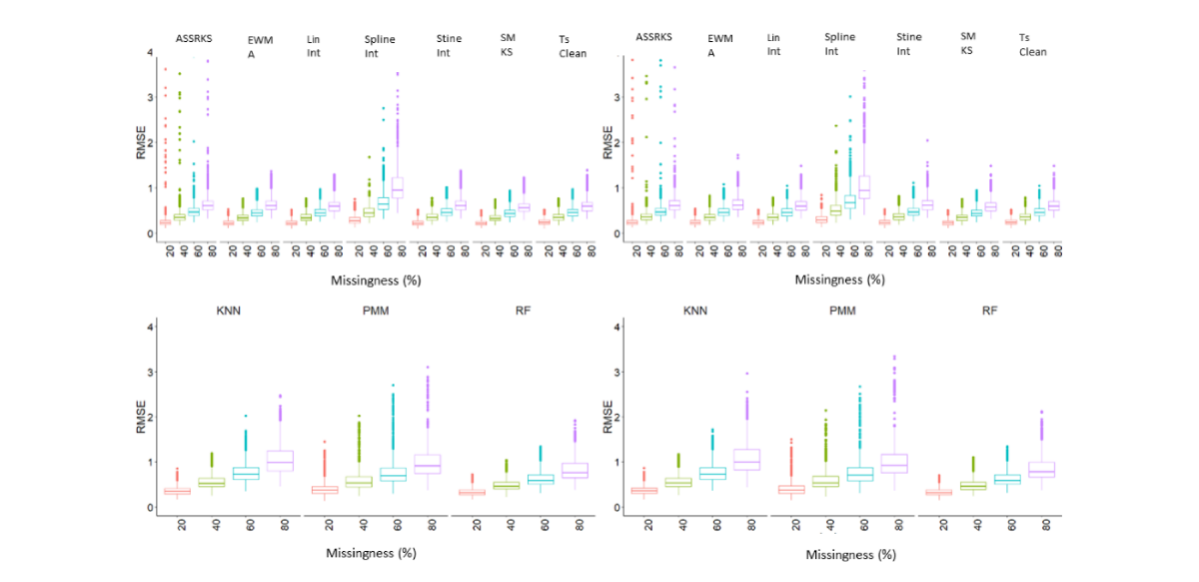
Illustration of linear and non-linear calculation of body weight variability. Scatterplots represent an example of a single participant with a non-linear weight trajectory over 12-months. Figure (A) shows a linear trendline fitted to the data with (B) the trendline subtracted and the associated residuals plotted. Figure (C) shows a non-linear locally estimated scatterplot smoothing regression fitted to the data with (D) the trendline subtracted and the associated residuals plotted. RMSE: root mean square error.

The results were grouped by imputation strategy and proportion of missingness. A summary of the performances is illustrated using the RMSE in Figure 3, and the full results are provided in Multimedia Appendix 4. To further test the imputation performance, we used the mean absolute percentage error and mean absolute error, the results of which are shown in Multimedia Appendix 5. The errors increased with greater amounts of missing data. SMKS showed the lowest errors overall, followed by EWMA, linear interpolation, and Stine interpolation, though each of these methods were similar in performance. Machine learning–based methods (RF and KNN) generally performed worse than univariate methods, as did the regression-based multivariate method PMM. The ASSRKS method showed the greatest error, followed by the spline interpolation. Imputation of MCAR-simulated data sets generally showed lower errors than RPM-simulated data sets.

### Calculation of BWV

**Figure 4:**
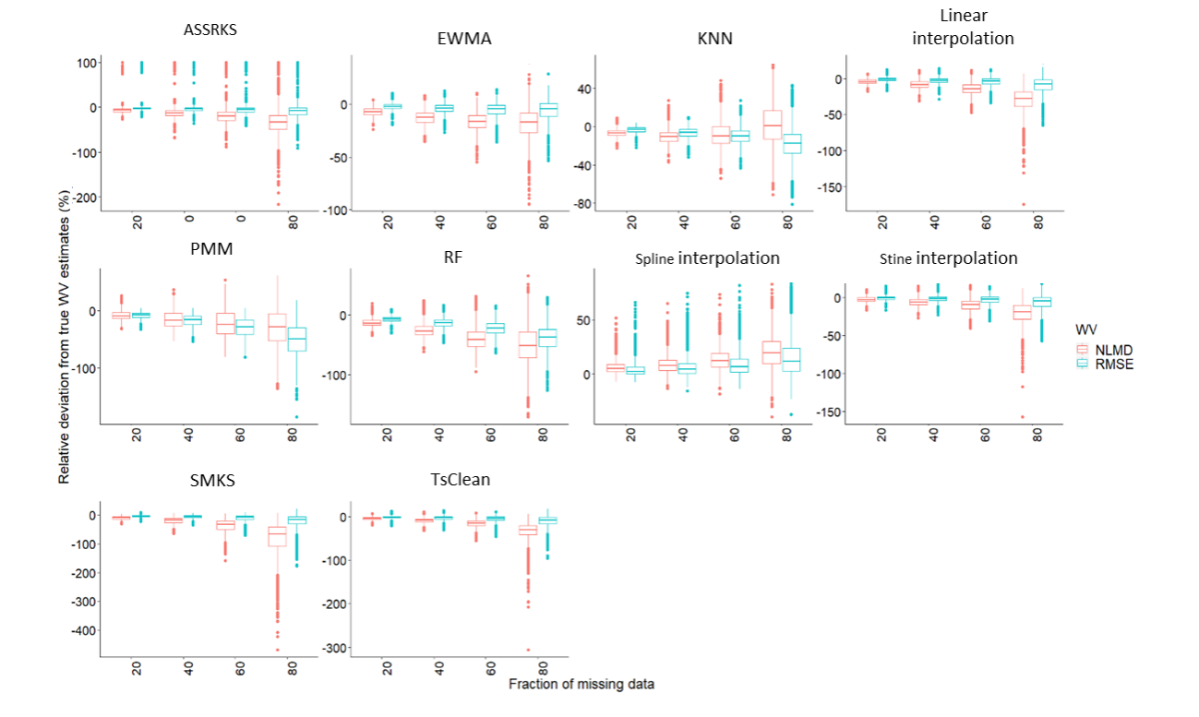
Influence of data imputation on linear and non-linear body weight variability estimates. Caption: Boxplots of the relative errors associated with calculation of body weight variability in body weight data collected by smart scales when using 10 different imputation methods imputing data in increments of 20%, 40%, 60%, and 80%. Errors represent the deviation from estimates made from observed data sets. ASSRKS: ARIMA state-space representation and Kalman smoothing; EWMA: exponentially weighted moving average; KNN: K-nearest neighbors; NLMD: nonlinear mean deviation; PMM: predictive means matching; RF: random forest; RMSE: root mean square error; SMKS: structural modeling with Kalman smoothing.

Next, we investigated the agreement between BWV estimations from observed data sets and simulated and imputed data sets for each participant. First, data sets simulated by MCAR and RPM were combined. For simulated data sets (ie, those with missing data), the errors were minimal, reaching an average of 7% (SD 15.4) and 3.2% (SD 19.5) disagreement between the true weight variability (WV) estimates and estimates made on 80% missing data for nonlinear and linear BWV calculation methods, respectively. At 60%, 40%, and 20% of missing data, errors were 2.3% (SD 9.1) and 0.6% (SD 7.3), 1.3% (SD 6.4) and 0.4% (SD 9.8), and 0.4% (SD 6.9) and 0.2% (SD 6.0) for nonlinear and linear WV estimates, respectively, compared with true estimates. The full results can be viewed in Multimedia Appendix 6. When data were imputed, imputation introduced substantial errors in BWV estimates (Figure 4). For most methods, imputation resulted in underestimation of BWV, apart from spline imputation, which overestimated BWV. Biases increased with missingness and were generally greater for NLMD than for RMSE.

## Discussion

In this study, we proposed a method for processing body weight data acquired from electronic smart scales, with both general and specific applications (to BWV). The analysis was produced in response to the increasing use of smart scales in clinical and research environments [24-26]. For the purposes of cleaning, imputation, and detrending, this analysis can inform most researchers dealing with body weight data from smart scales. Furthermore, we provide specific validations on the estimation of BWV using linear and nonlinear approaches and report the errors associated with these estimations when (1) data are missing and (2) data are imputed. We found that SMKS, EWMA, and linear interpolation performed imputation best. These methods are available to researchers through many statistical packages [39]. For the purpose of estimating BWV, we showed that leaving data as missing does not introduce significant bias (only 3%-7% error with >80% data missing), whereas calculating BWV on imputed data causes significant underestimation and should be avoided.

### Body Weight Imputation

We considered 7 univariate and 3 multivariate approaches to imputation. As access to further individual-level information (eg, participant characteristics or behavioral patterns and psychological traits) may be unavailable, body weight data collected by smart scales may be treated as univariate, and therefore, the use of more advanced approaches to multivariate imputation such as tree-based models, neural networks, and KNN methods is limited. To test multivariate imputation algorithms, we added day number (ie, day of trial) and day of the week as predictive variables, as these can be automatically collected in free-living environments without any participant burden. Within-week (eg, weekday vs weekend) fluctuations in body weight have been shown previously [19,20] and may have predictive value in imputation. However, we found that these methods, in the current circumstances, did not outperform simple methods such as SMKS or EWMA on MCAR- or RPM-simulated data sets. Indeed, machine learning methods may perform better when trained on large, complete data sets and then applied to missing data; however, in this analysis, we did not have enough complete data sets to train machine learning imputation models, and we chose to limit the variables used in multivariate imputation to improve accessibility. This is the first study to address the issue of missingness and imputation in body weight tracking data; however, several studies have addressed the tracking of physical activity from accelerometers [25,61,62], often using similar simulation and validation approaches with success.

### Body Weight Variability Estimation

We proposed a method of estimating BWV using a nonlinear approach, which we termed NLMD. This was devised to address the assumption of linearity associated with RMSE estimations commonly used. Using a nonlinear approach, the trendline is fitted more closely to the data. The result is the ability to identify day-to-day variability or within-week patterns. In contrast, in the case of curvilinear weight trends, RMSE generates large residual errors; this may be more suitable when the aim is to detect larger fluctuations over several months or years. We found that BWV estimates from data sets with simulated missingness were similar to true estimates, using both RMSE and NLMD methods. Surprisingly, using our current methods, BWV estimates were not greatly different between complete and 80% missing data sets (3.2%, SD 0.2% and 7.0%, SD 0.2% for RMSE and NLMD methods, respectively). However, when these missing data were imputed, substantial biases were introduced largely as underestimations, which increased for each increment of imputed data. As such, although our imputation-validation analysis may inform general imputation of body weight data for numerous other purposes, for the purpose of estimating BWV, we advise that data be left as missing.

To our knowledge, no previous study has examined long-term BWV from electronic smart scales, and only a few studies have modeled frequent weight data using TS methods. A recent study examining the effect of breaks in self-weighing on weight outcomes used a linear mixed model approach using time and weight as fixed predictor and response variables, respectively [37]. However, the use of linear modeling when examining BWV is not sensitive to the often-polynomial features of body weight trajectories. In another study, the authors compared differences in weekday and weekend body weights with longer-term weight changes [20]; however, the data were not detrended. Therefore, weekly weight patterns may potentially be sensitive to overall weight change (particularly in individuals with rapidly changing weight). In a study examining the effect of season on weight patterns across several countries, the authors fitted orthogonal polynomials to the weight data before conducting a detrending process, which may help isolate seasonal patterns from the overall trend (eg, loss or gain) of an individual, showing clear seasonal patterns across the year in different geographical regions [23]. Finally, in a recent study investigating within-week patterns of BWV in 80 adults, the authors took a comprehensive approach by applying nonparametric smoothing techniques (similar to this study) and removed the trend component of the TS using a moving average approach, reporting significant weekly patterns within a week characterized by weekend weight gain and weekday compensation [19]. Recently, we used the present methods to inform the description of weight fluctuation patterns across weeks, years, and holidays [1] and to investigate the associations between BWV and cardiometabolic health outcomes [2].

### Strengths and Limitations

This study has several strengths. First, we developed our data processing methods from true rather than simulated data, thus increasing the validity of the analysis. Our simulation-imputation analysis was comprehensive, including the generation of 8000 missingness-simulated data sets in total with varying levels of missingness using both random and real-missingness informed simulations, which resulted in 80,000 imputed data sets produced using 10 univariate and multivariate algorithms. Next, we described and compared both linear and nonlinear approaches to estimating BWV under different conditions of missingness and reported the errors produced in the common case of missing data, which should inform the magnitude of errors expected from missing data estimations in future studies. Some limitations should also be addressed. First, all imputation methods were deterministic, although body weight seems to be a relatively stochastic (ie, randomly determined) process. The resultant effect is that imputation may reduce the variability by attempting to recognize predictive patterns that are not there. We recommend that consideration should be given to whether imputation is necessary. In some analyses, including instances where machine learning algorithms are employed, complete data are a necessity; therefore, imputation is required. Next, we did not have entirely complete data by which to test imputation, although we opted to use real rather than simulated data for external validity.

### Conclusions

BWV potentially represents (1) a significant health risk and (2) a prognostic tool that is currently not well understood or well measured. This study evaluated the performance of various imputation methods applied to body weight data and presented a protocol for estimating BWV under varying amounts of missing data. We showed that structural modeling with a Kalman smoother and EWMA performed an imputation most effectively. However, in the case of estimating BWV, the imputations generally produced large underestimations due to the tendency to revert toward the mean. Furthermore, we demonstrated the errors associated with BWV estimates at varying levels of missing data, concluding that errors are small when using both linear and nonlinear methods even under high proportions of missingness. In future, the importance of both frequent measurement of body weight and consistent and appropriate methods of analyzing the data produced should be underlined in the study of BWV.

## Appendix

Multimedia Appendix 1 Outlier detection limits based on physiological plausibility.

Multimedia Appendix 2 Detailed description of the data removal processes.

Multimedia Appendix 3 Illustrated examples of body weight imputation by all methods.

Multimedia Appendix 4 Table of imputation performance by root mean square error.

Multimedia Appendix 5 Summary figures of imputation performance by mean absolute percentage error and mean absolute error.

Multimedia Appendix 6 Table of mean errors in body weight variability calculation following simulation and imputation.

## Abbreviations

ARIMA: AutoRegressive Integrated Moving Average
ASSRKS: ARIMA state-space representation and Kalman smoothing
BWV: body weight variability
EWMA: exponentially weighted moving average
KNN: K-nearest neighbors
LOESS: locally estimated scatterplot smoothing
MAR: missing at random
MCAR: missing completely at random
NLMD: nonlinear mean deviation
PMM: predictive means matching
RCT: randomized controlled trial
RF: random forest
RMSE: root mean square error
RPM: real patterns of missingness
SMKS: structural modeling with Kalman smoothing
TS: time series
WV: weight variability
