# Supplementary material for: Data Imputation and Body Weight Variability Calculation Using Linear and Nonlinear Methods in Data Collected From Digital Smart Scales: Simulation and Validation Study
Source: JMIR Mhealth Uhealth. 2020 Sep 11;8(9):e17977. doi: 10.2196/17977 (PMC7519428; doi:10.2196/17977)
Supplement: Multimedia Appendix 1 [file mhealth_v8i9e17977_app1.docx]

Data Imputation and Body Weight Variability Calculation Using Linear and Nonlinear Methods in Data Collected From Digital Smart Scales: Simulation and Validation Study

Multimedia Appendix 1

| Limits of weight change within a given time period | |
| --- | --- |
| Change in body weight | Duration |
| ± 5% | 1 week |
| ± 10% | 4 weeks |
| ± 15% | 8 weeks |
| ± 20% | 12 weeks |

Maximum limits of weight change allowed, outwith which data are removed as outliers. Limits are informed by physiologically plausible observations under conditions of substantial under or overfeeding, as referred to in-text.
