# Supplementary material for: Data Imputation and Body Weight Variability Calculation Using Linear and Nonlinear Methods in Data Collected From Digital Smart Scales: Simulation and Validation Study
Source: JMIR Mhealth Uhealth. 2020 Sep 11;8(9):e17977. doi: 10.2196/17977 (PMC7519428; doi:10.2196/17977)
Supplement: Multimedia Appendix 2 [file mhealth_v8i9e17977_app2.docx]

Data Imputation and Body Weight Variability Calculation Using Linear and Nonlinear Methods in Data Collected From Digital Smart Scales: Simulation and Validation Study

Multimedia Appendix 2

In general, data can be missing from any data set following three broad mechanisms: missing completely at random (MCAR), missing at random (MAR) and not missing at random (NMAR). Absence of body weight data may have identifiable mechanisms, for example breaks in self-weighing may be indicative of weight gain [1], however these patterns may not be consistent between or within individuals. Data described as MCAR has no mechanism of missingness and can be considered completely random, however data which is MAR means that the missingness is not related to the missing data but may be partially explained by the observed data [2].

It is possible to test for the mechanism of missingness in a data set. Multivariate data sets where several variables are related provide greater ability to detect NMAR and MAR, though in univariate time series (such as body weight data) in many individuals, it is difficult to detect these mechanisms unless missing data is consistent at group level (e.g. all individuals don’t weigh themselves at weekends). With the present sample of participants, we tested for mechanisms of missingness using the TestMCARNormality function from the MissMech package [3] which reported that the data was MCAR. For this reason, we chose to insert data using MCAR methods at varying levels of missingness (20%, 40%, 60%, 80%) using the ImposeMissing function from the simsem package [4].

However, due to the potentially large variation in the patterns of missingness present in self-weighing data, we also made the decision to insert missingness which was identical to missing patterns in observed data from participants of The NoHoW trial. We termed this simulation mechanism real patterns of missingness (RPM). To do this, we selected 20 participants with approximately 20%, 40%, 60% and 80% missing data. Twenty was selected to ensure that we stayed around these set increments, whereas increasing this number would results in more variability around the increments (e.g. 15-25% etc). In doing this, the length of missing gaps, and structure of the gaps is more likely to represent that seen in true data. The following protocol was followed:

1. Select 20 participants with 20%, 40%, 60% and 80% missing data from the entire NoHoW trial group (80 in total).
2. Transform all 80 participants’ weight data to binary values representing present or missing.
3. Impose all 80 patterns of missing data on each of the 50 complete data sets (generating 4,000 simulated data sets).
4. Impute these data sets using univariate and multivariate methods
5. Test the performance of the imputation.
