# Supplementary material for: Data Imputation and Body Weight Variability Calculation Using Linear and Nonlinear Methods in Data Collected From Digital Smart Scales: Simulation and Validation Study
Source: JMIR Mhealth Uhealth. 2020 Sep 11;8(9):e17977. doi: 10.2196/17977 (PMC7519428; doi:10.2196/17977)

Data Imputation and Body Weight Variability Calculation Using Linear and Nonlinear Methods in Data Collected From Digital Smart Scales: Simulation and Validation Study

Multimedia Appendix 3

Illustrated examples of 7 univariate and 3 multivariate imputation techniques imputing data with 40% and 80% missingness inserted at random. A single participant with the greatest amount of data has been used as an illustrative example.


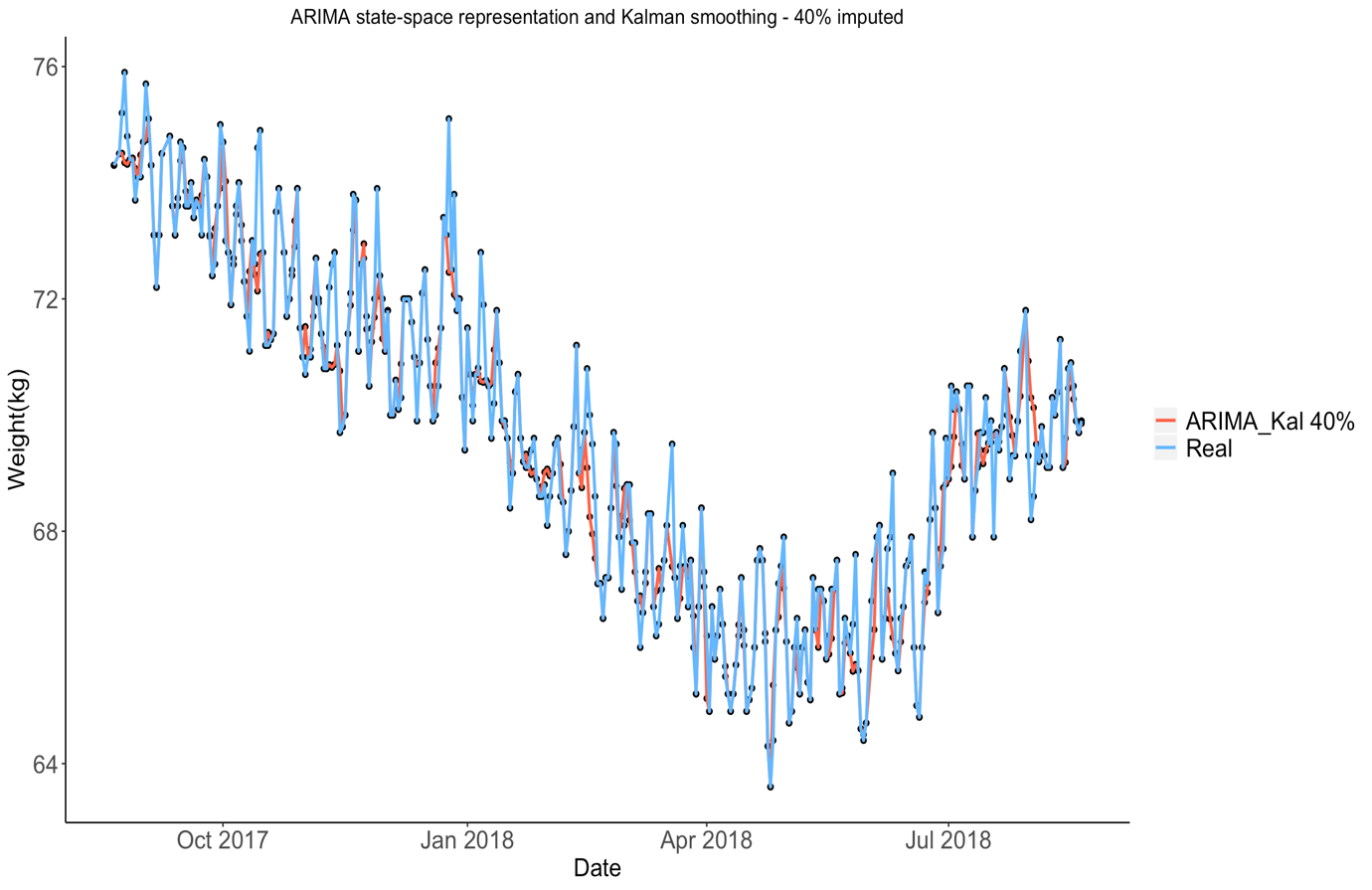


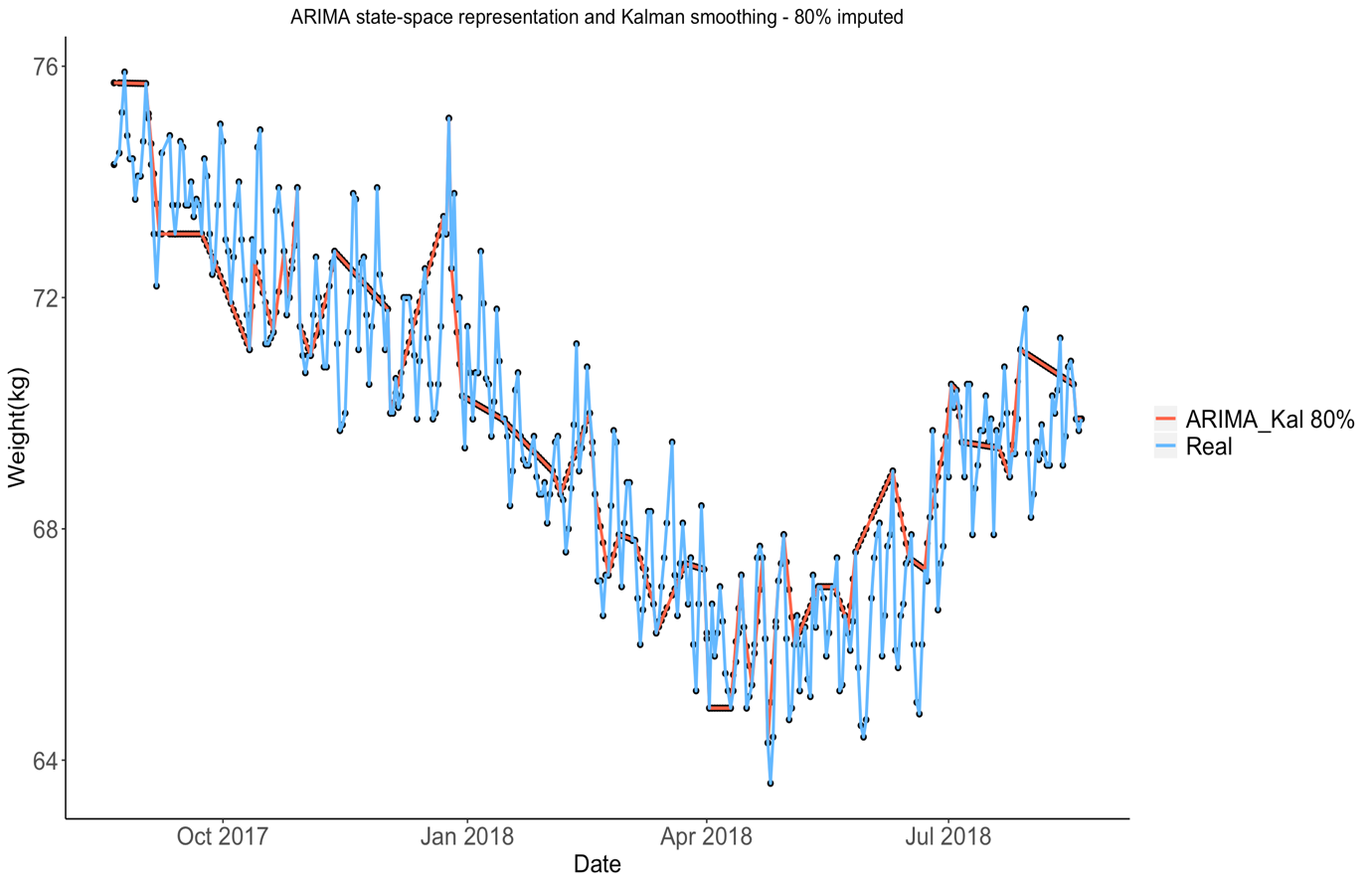


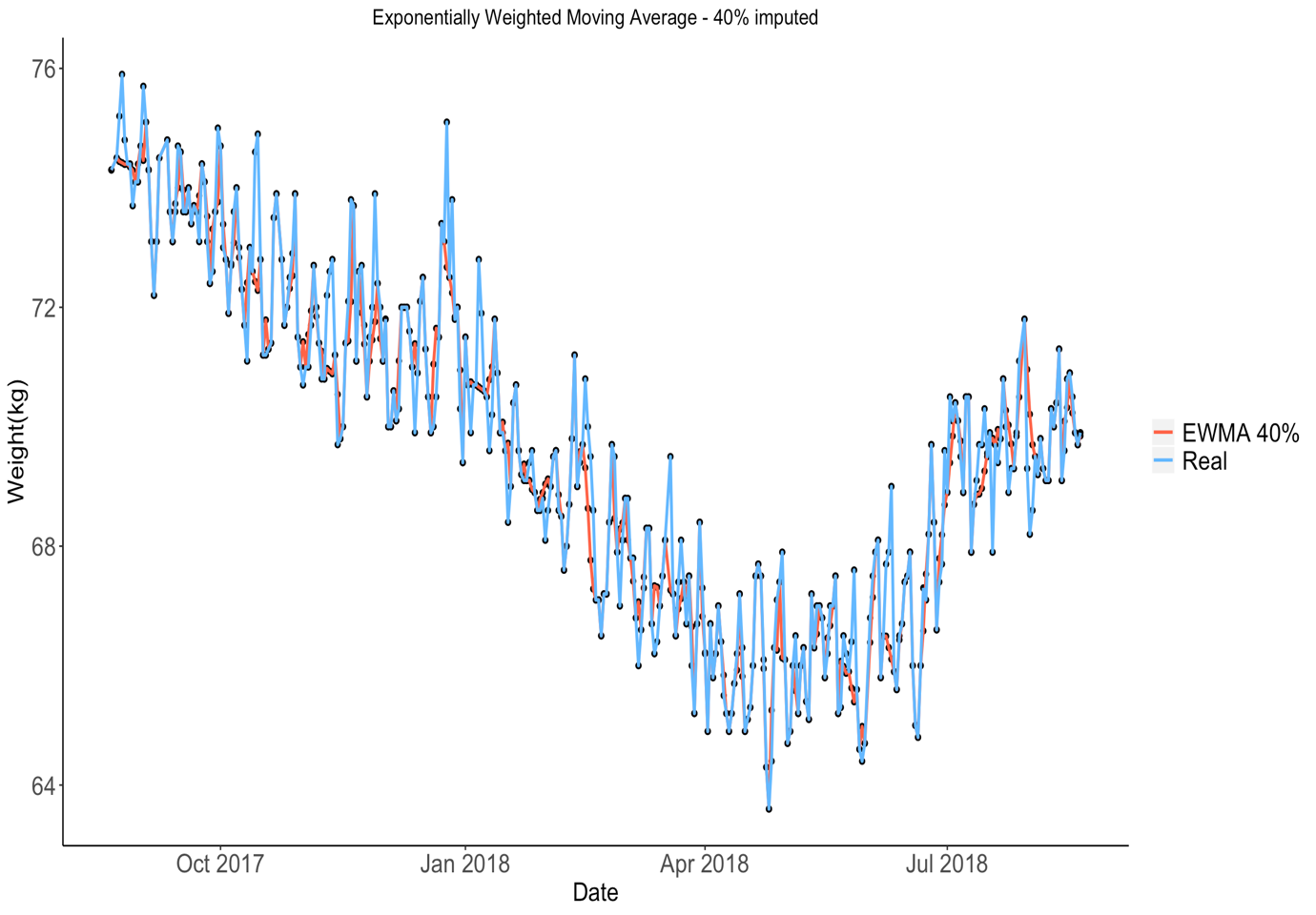


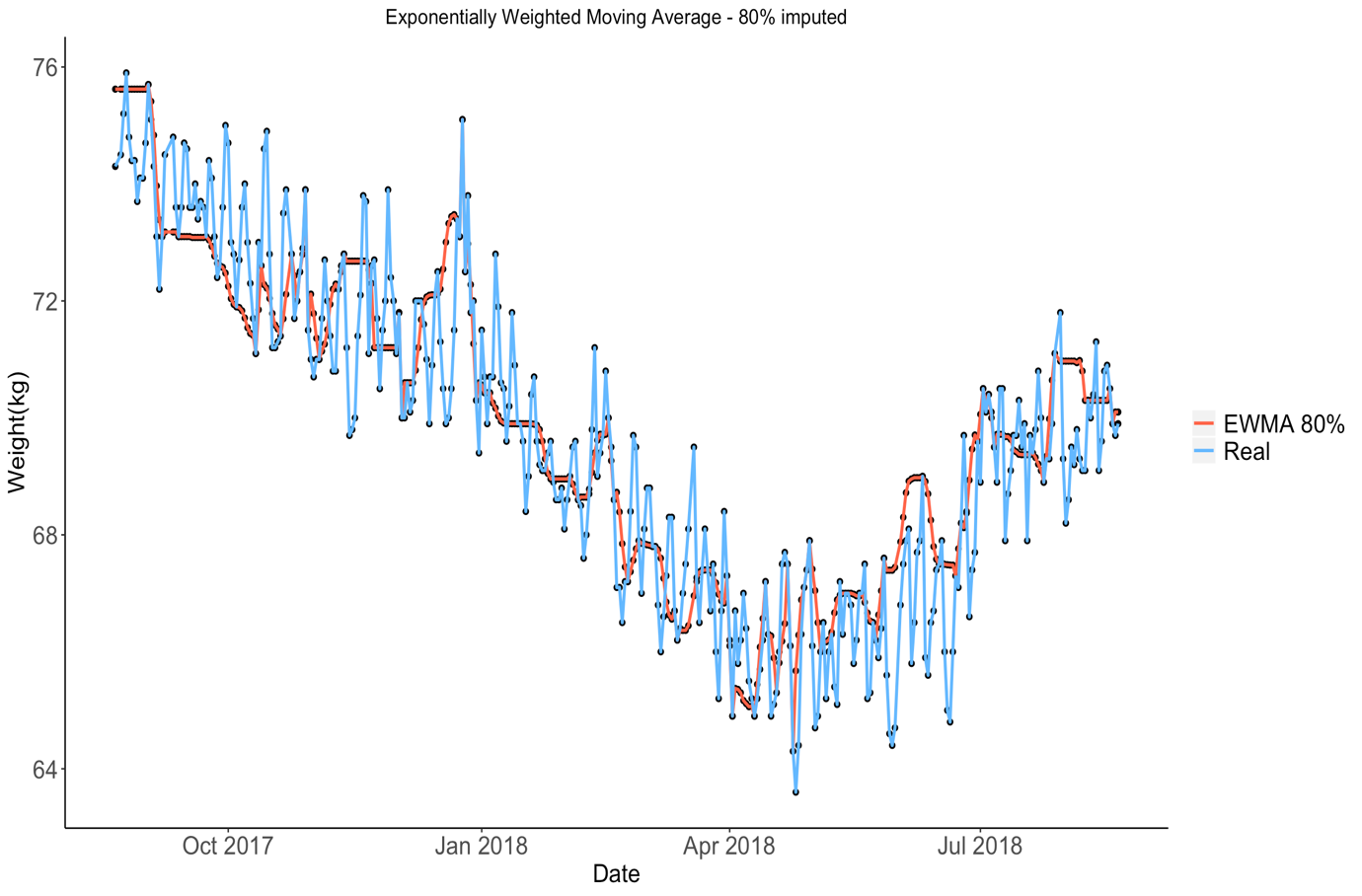


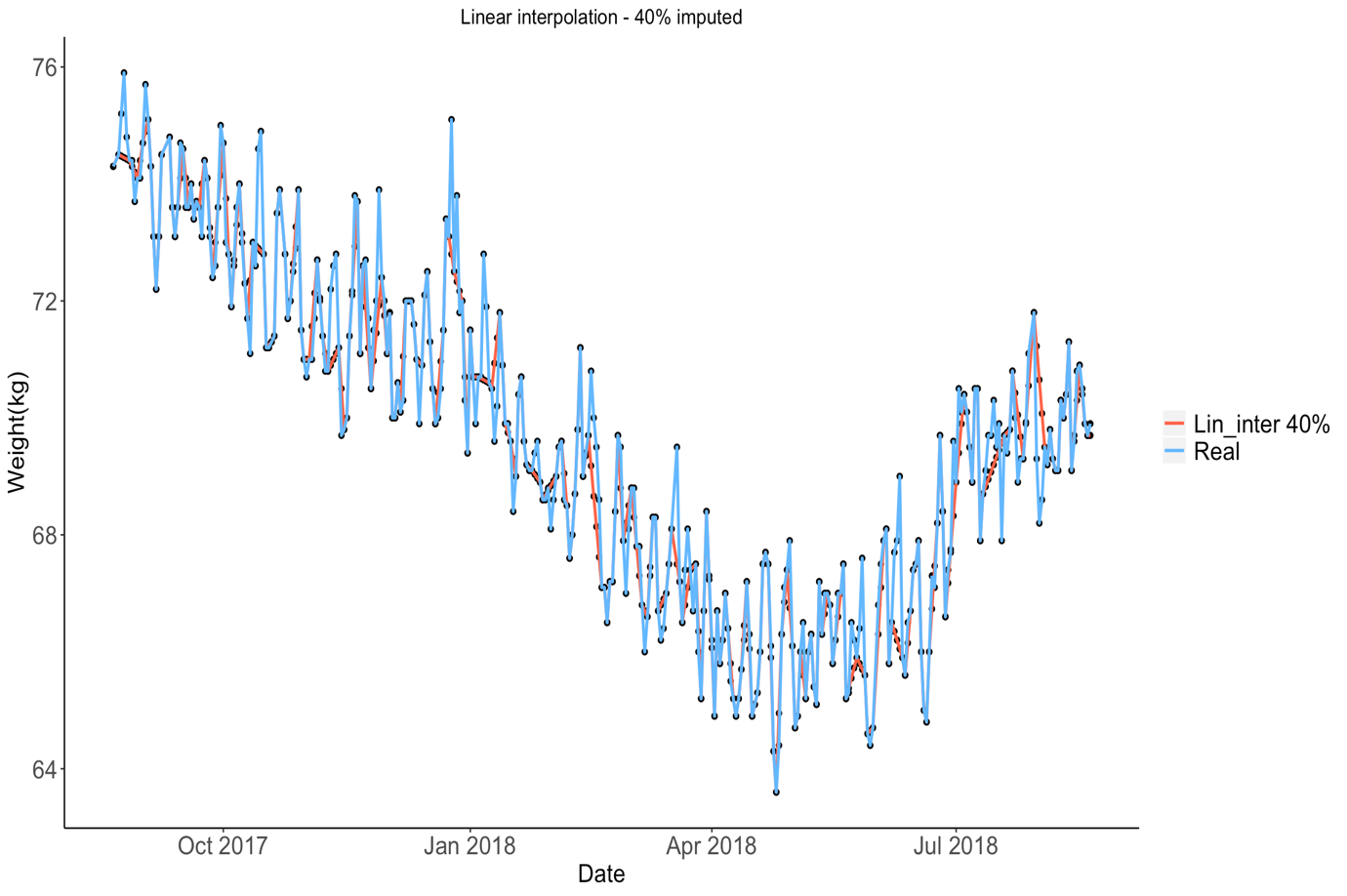


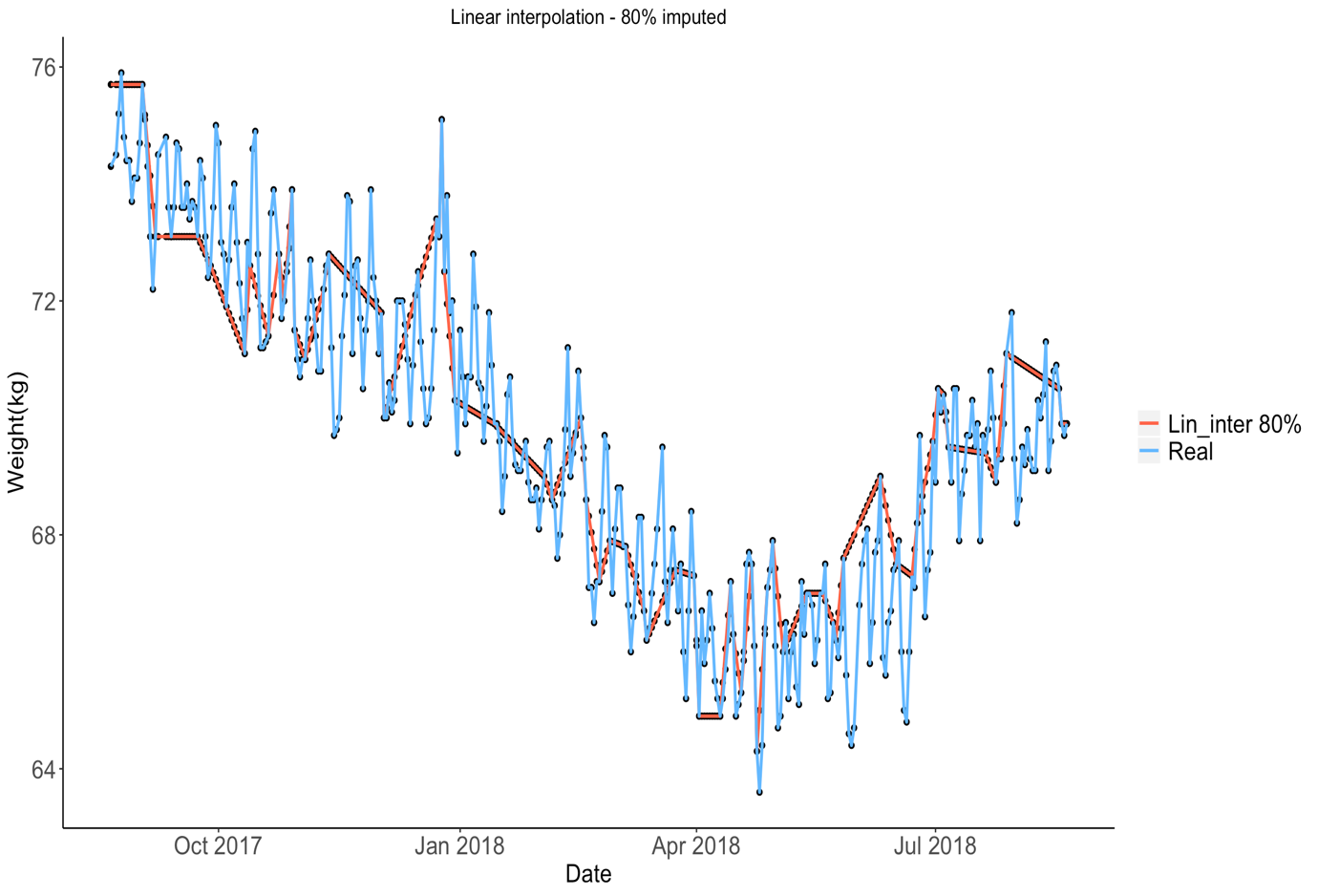


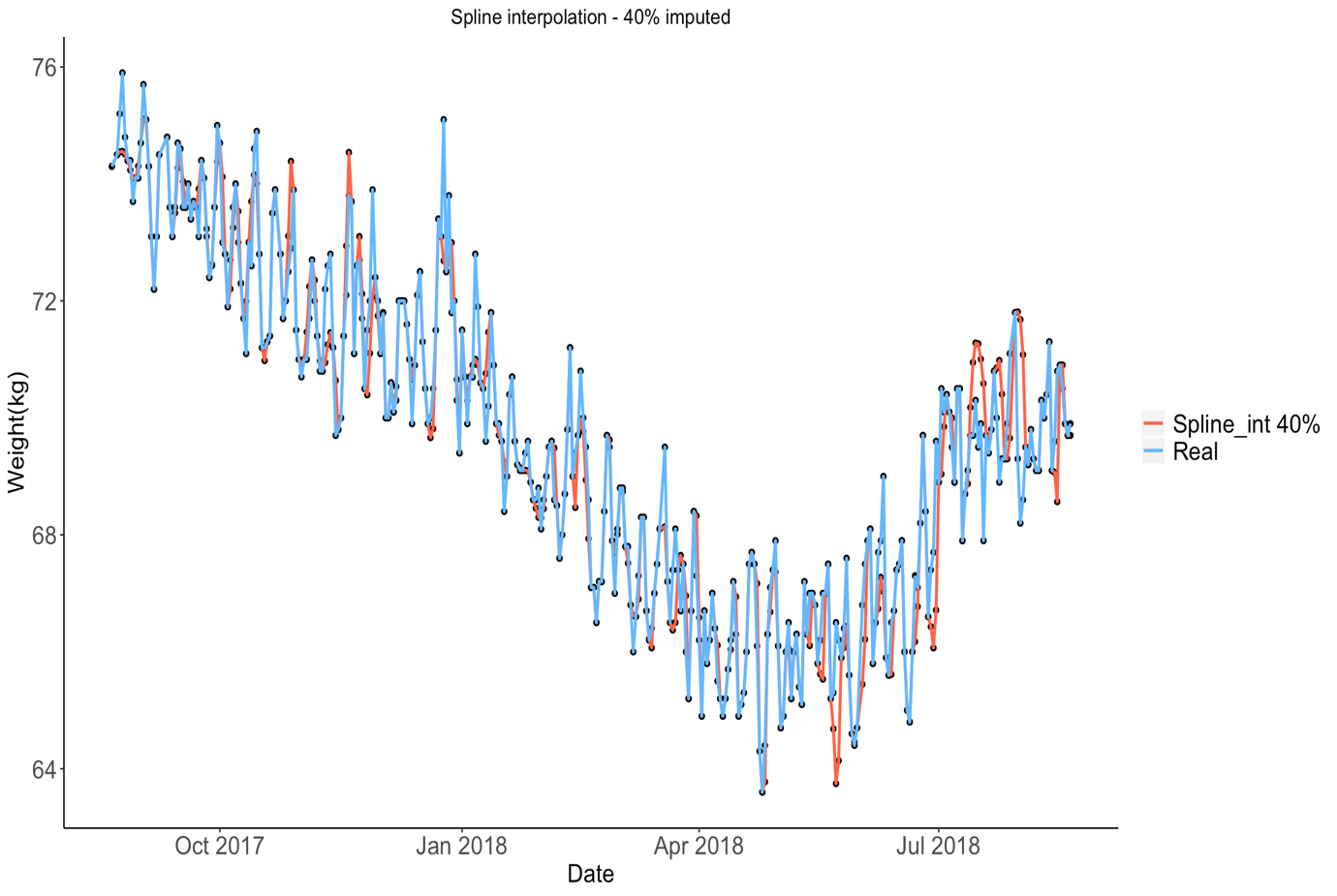


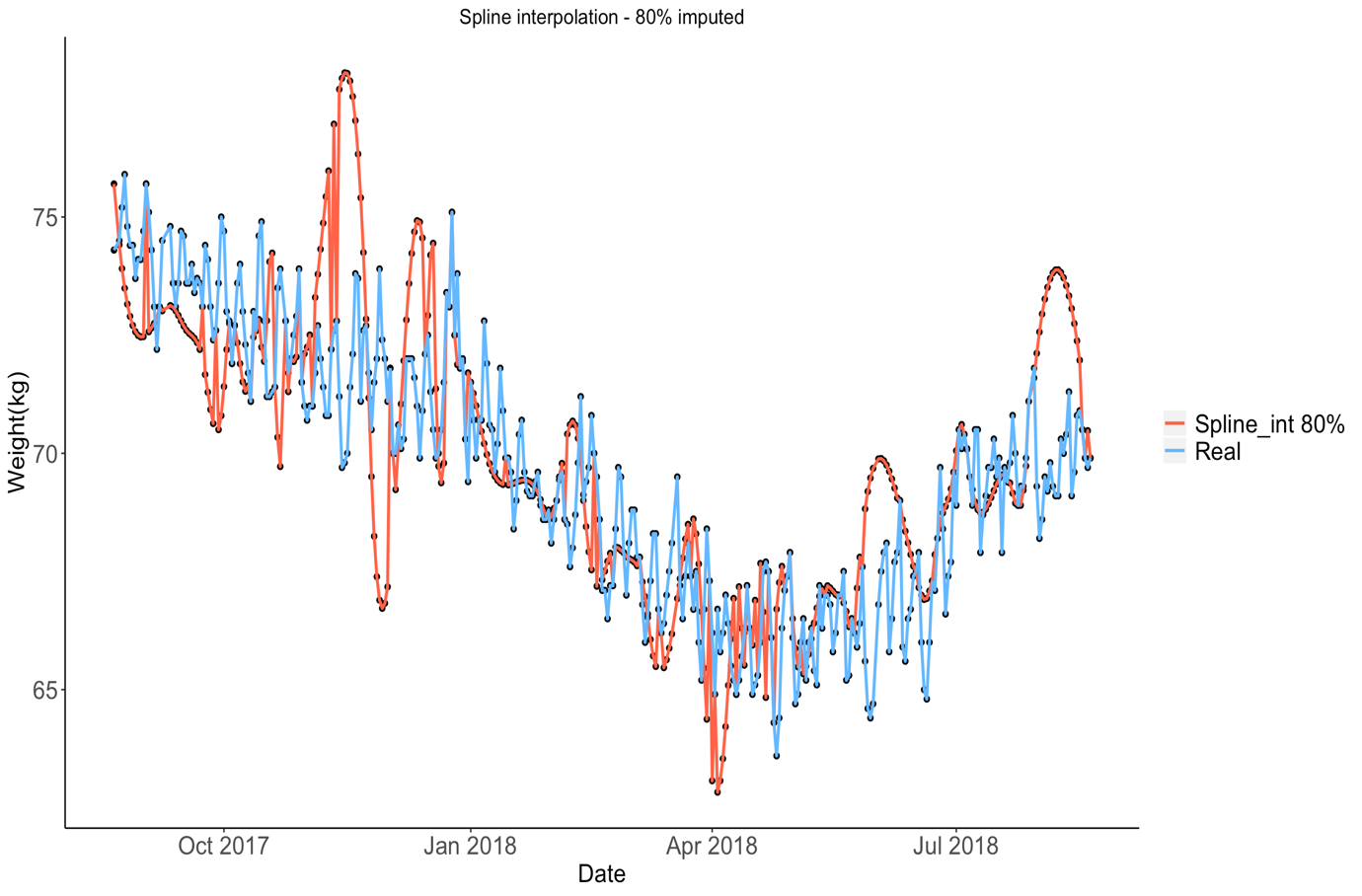


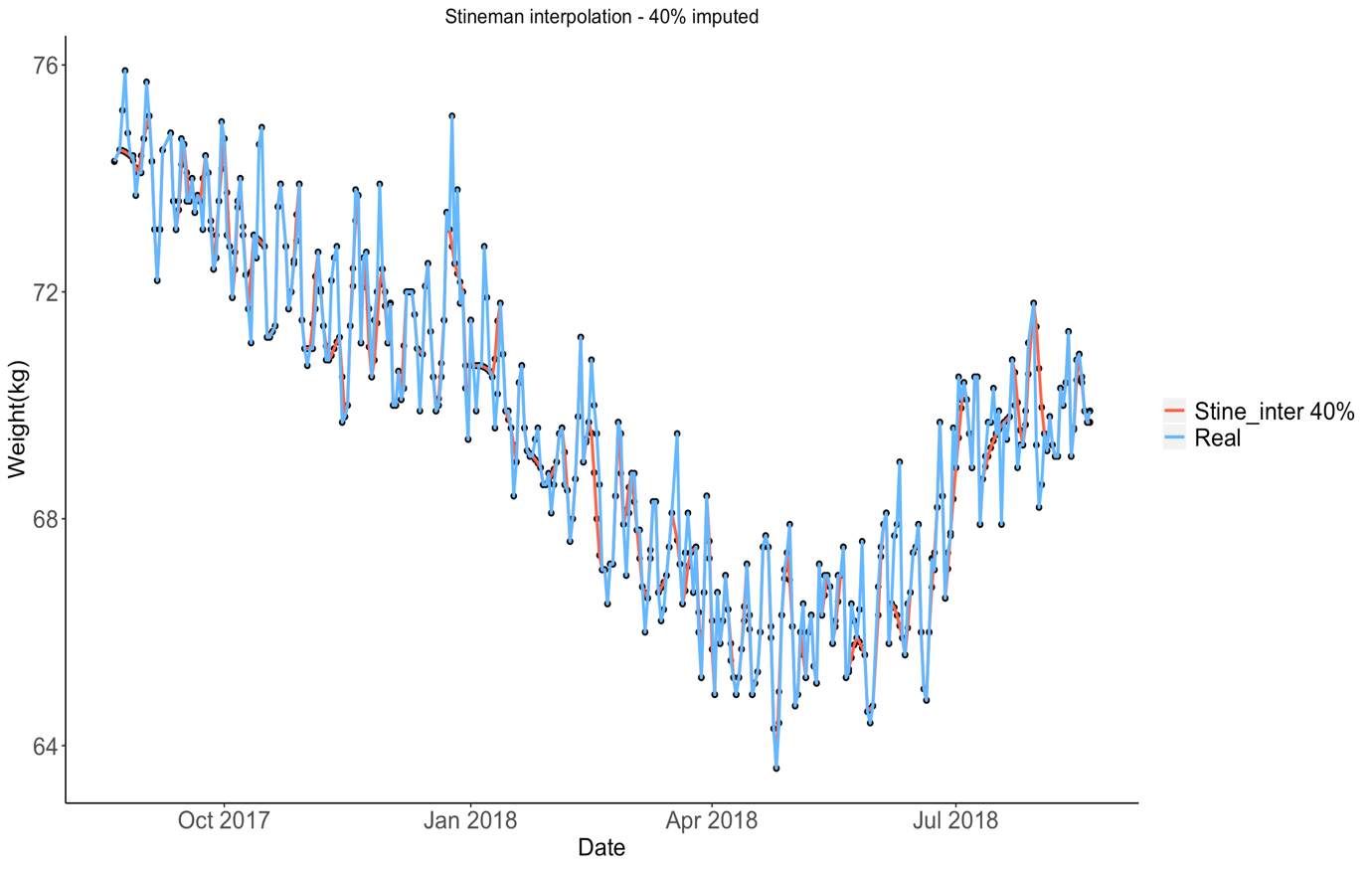


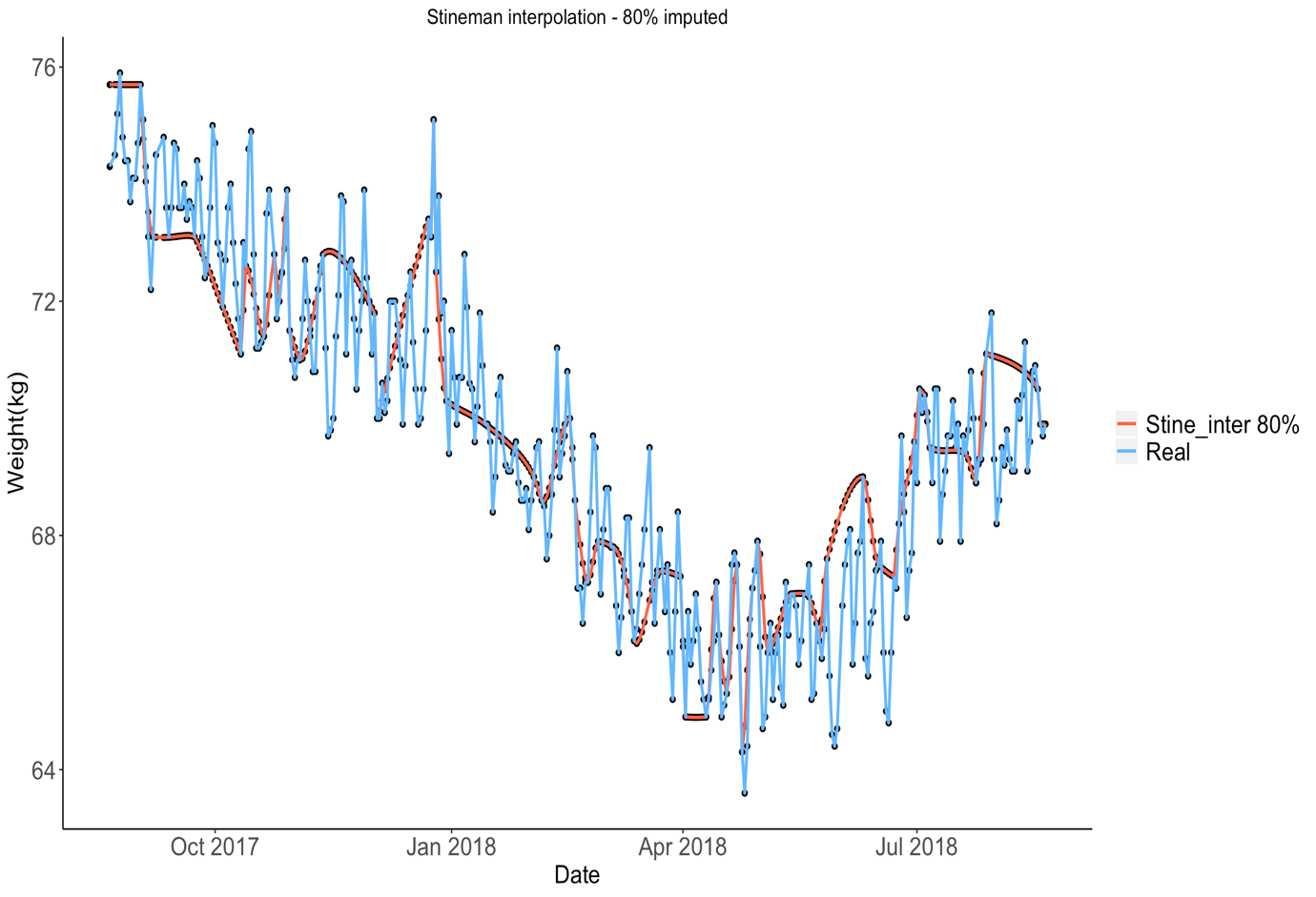


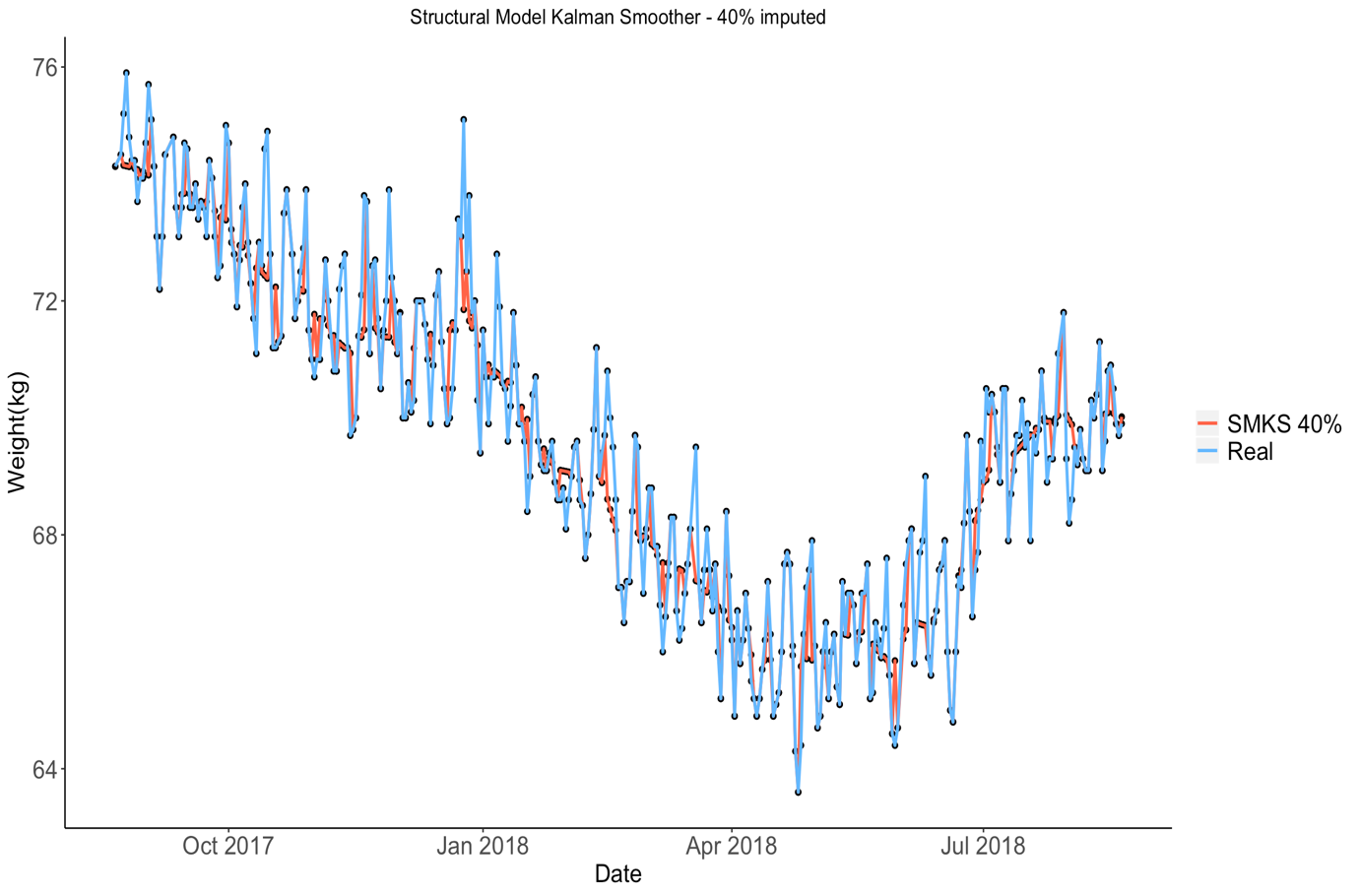


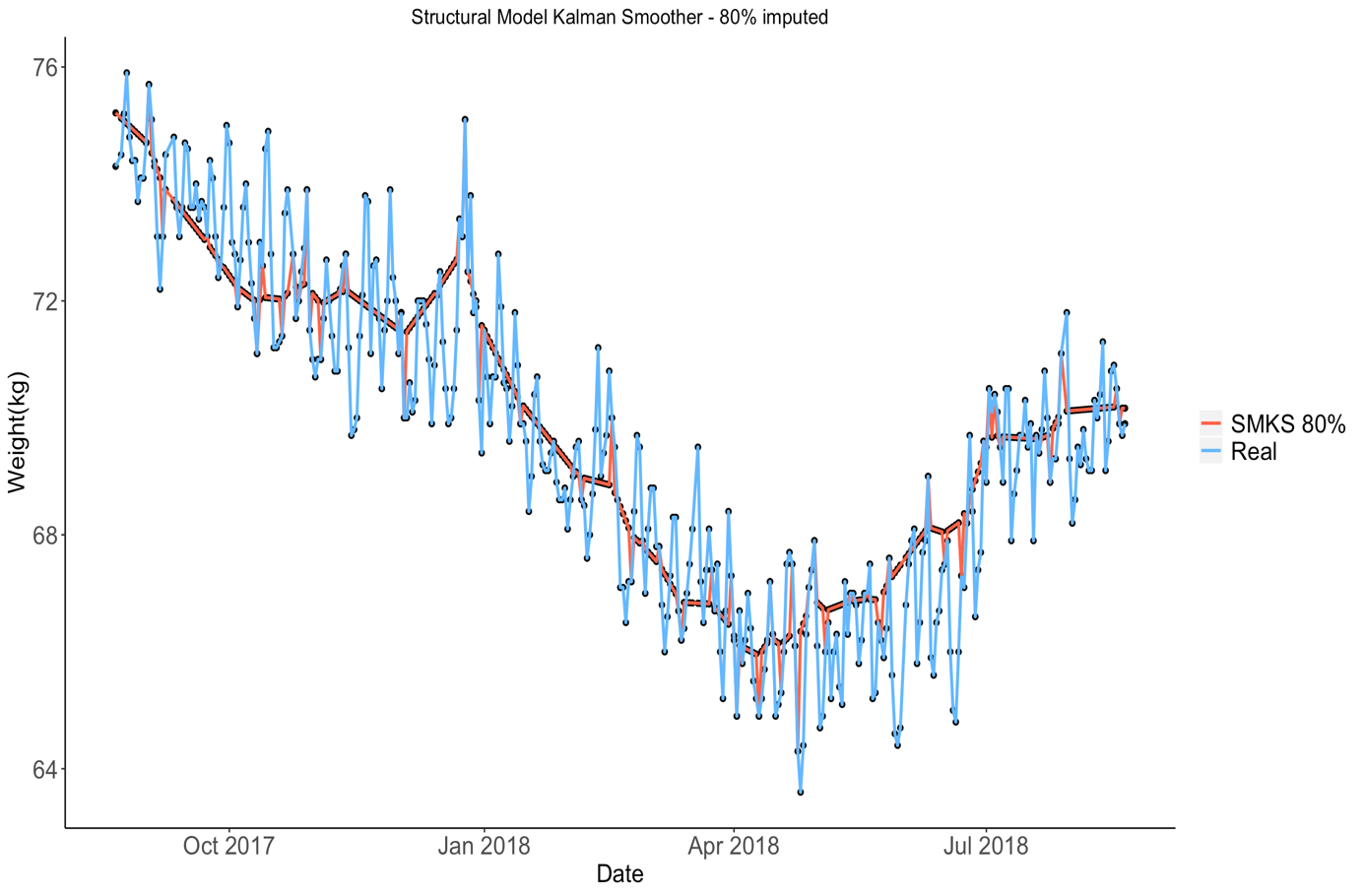


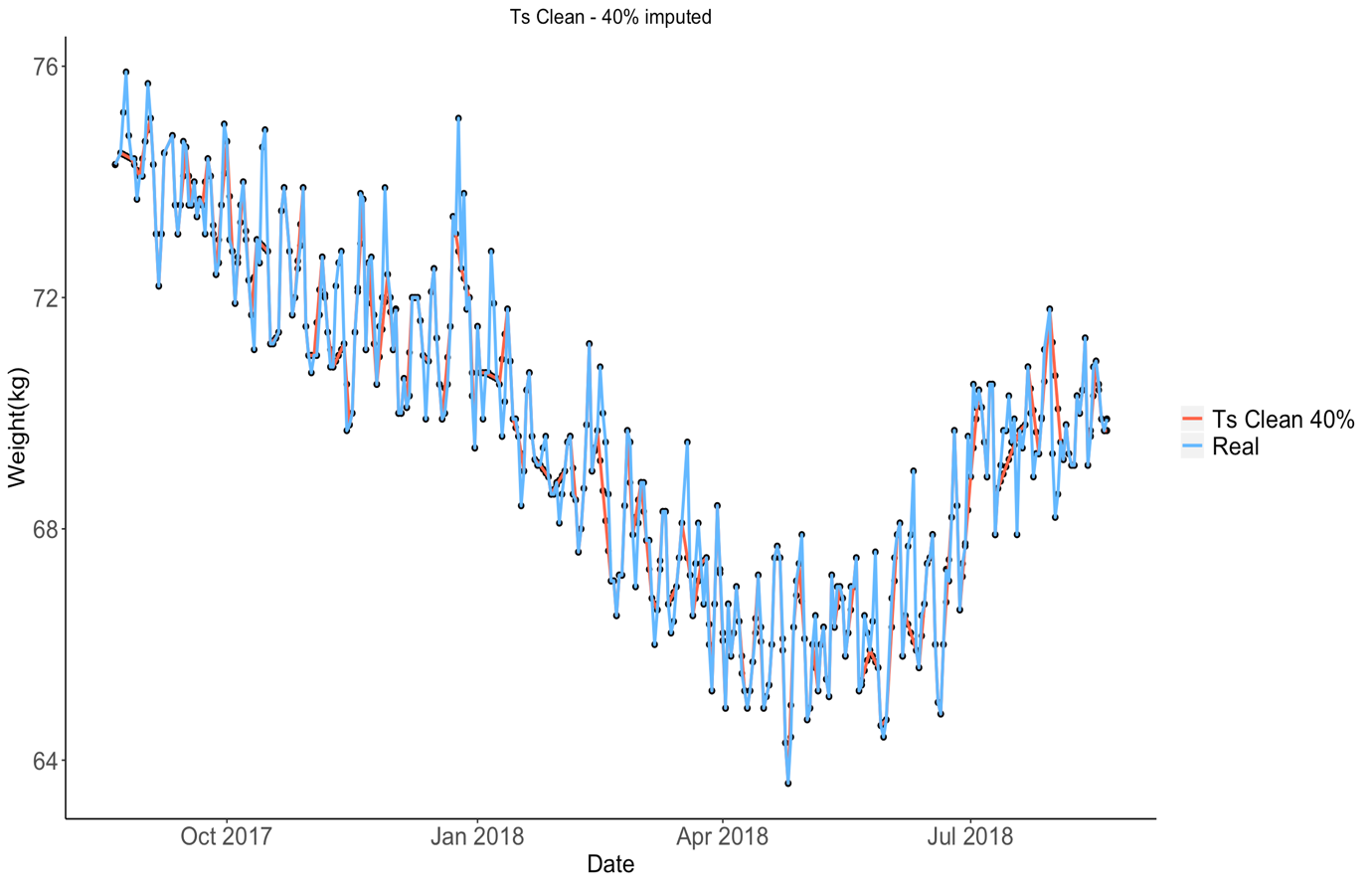


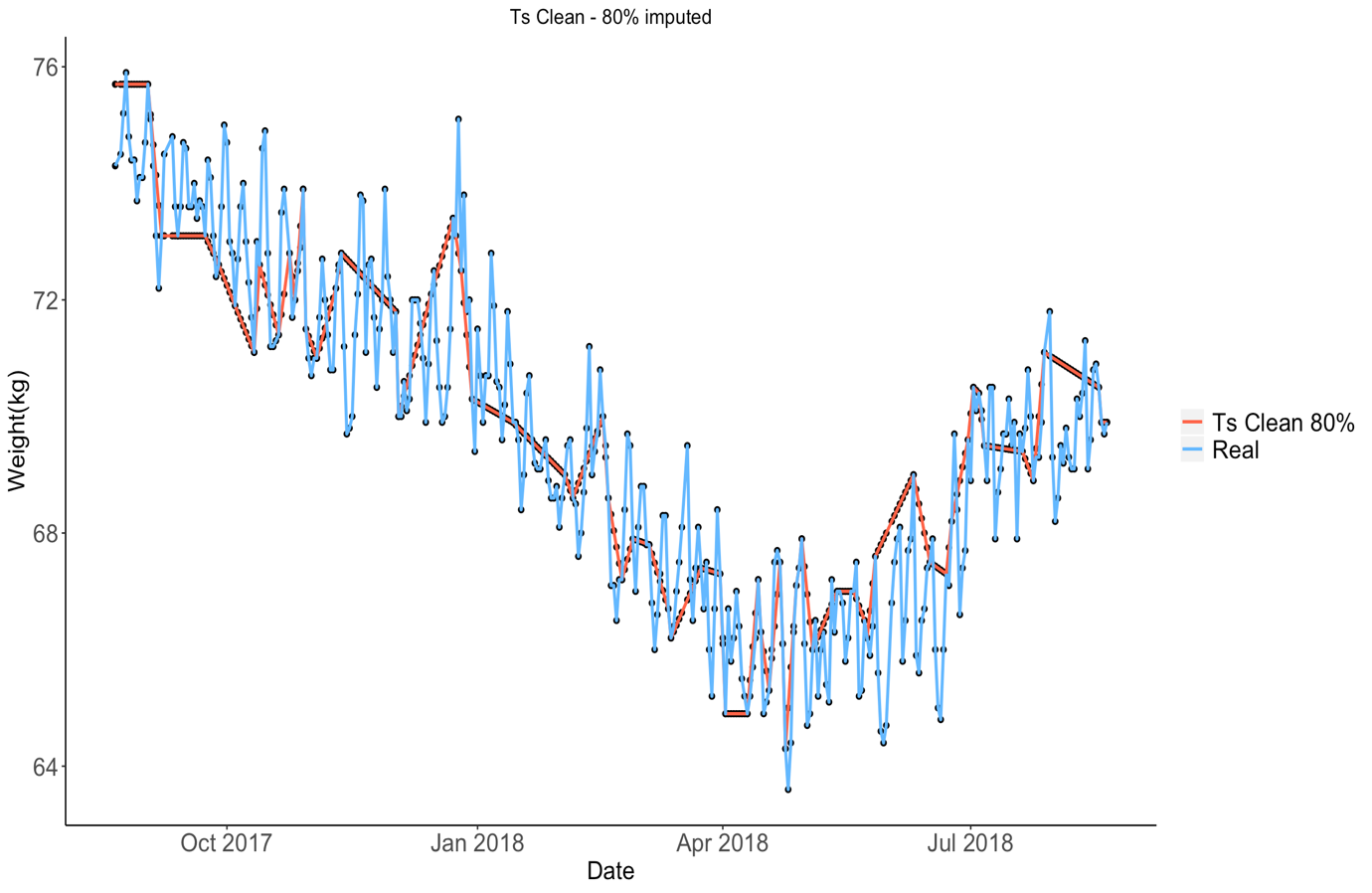


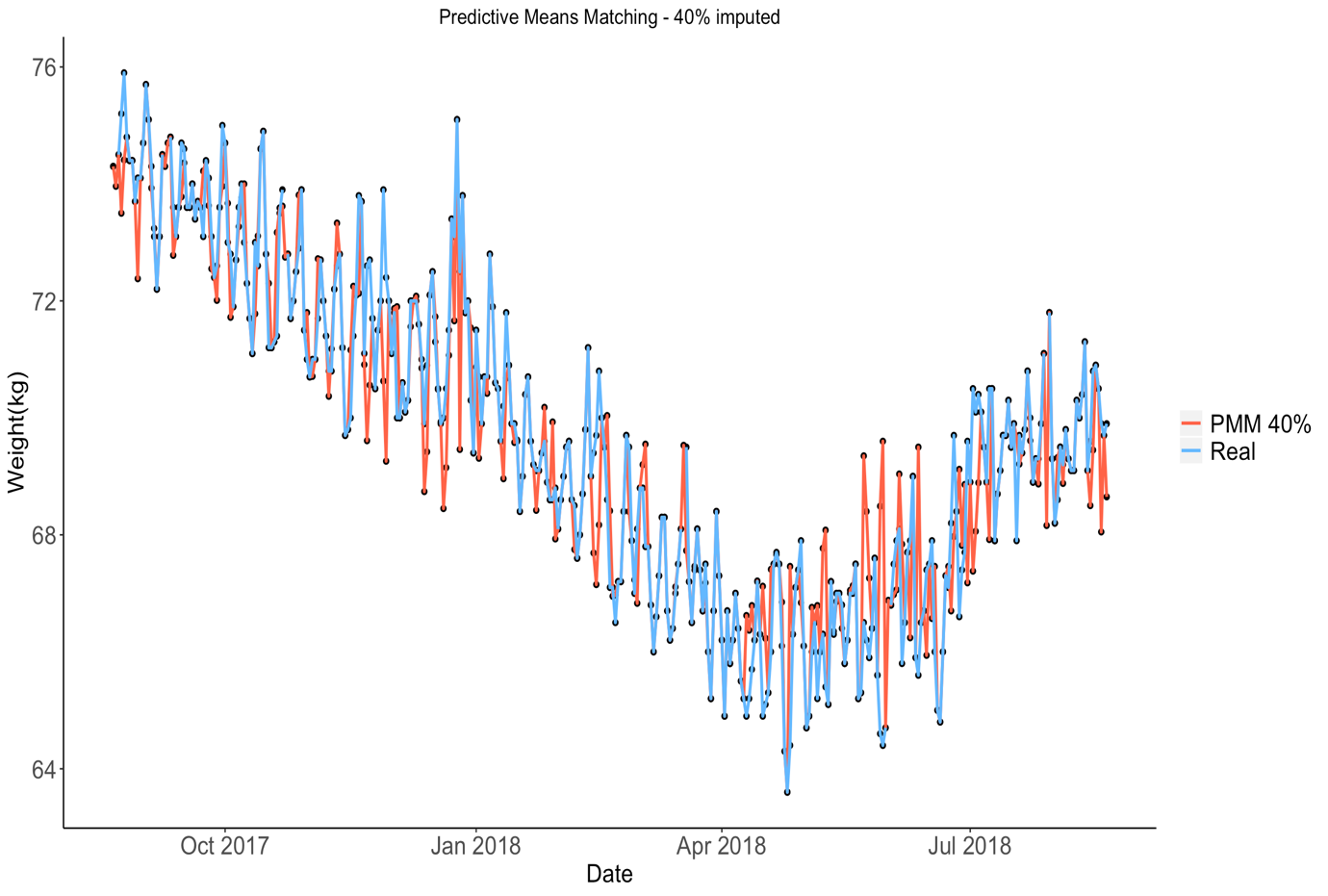


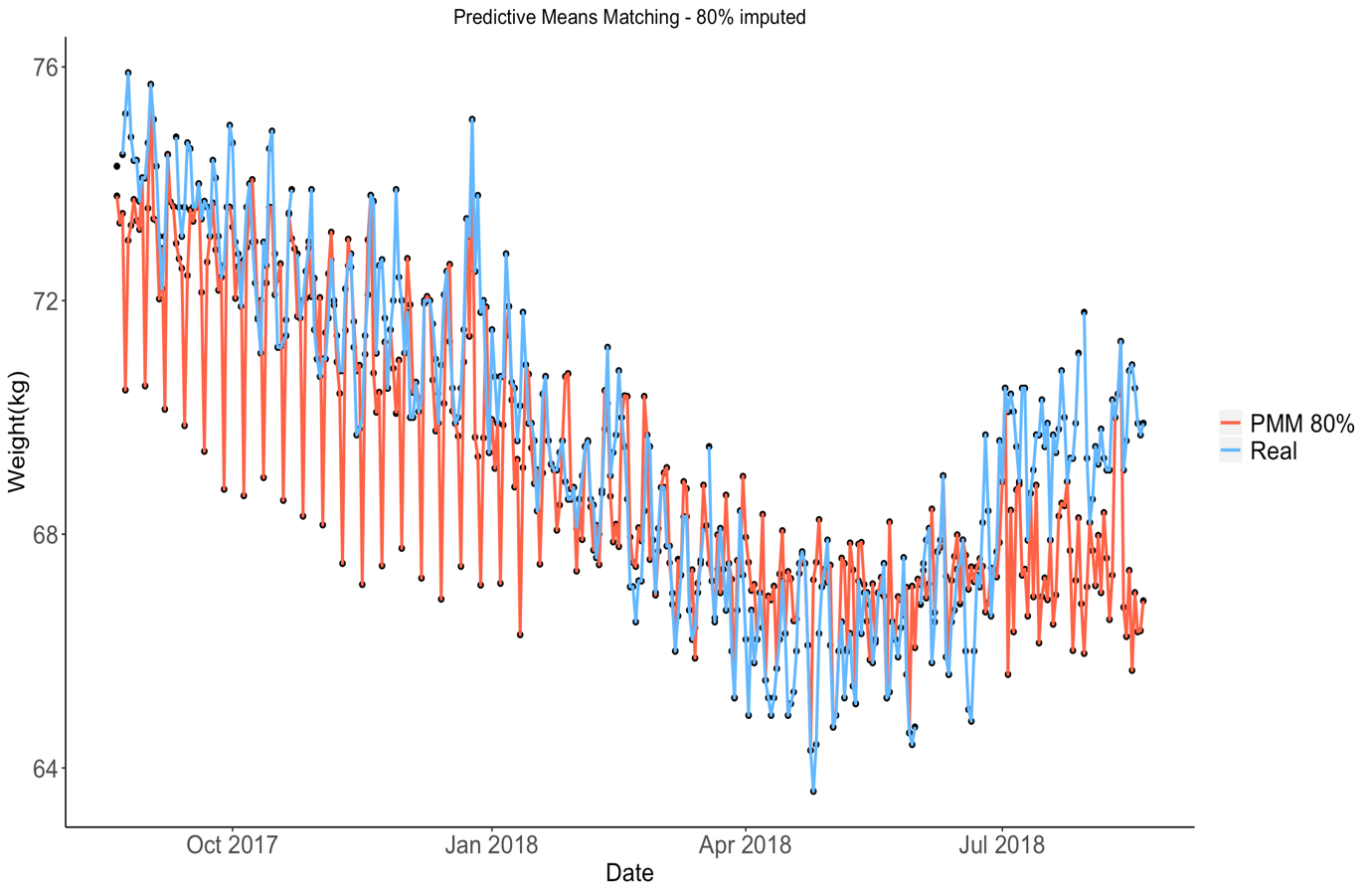


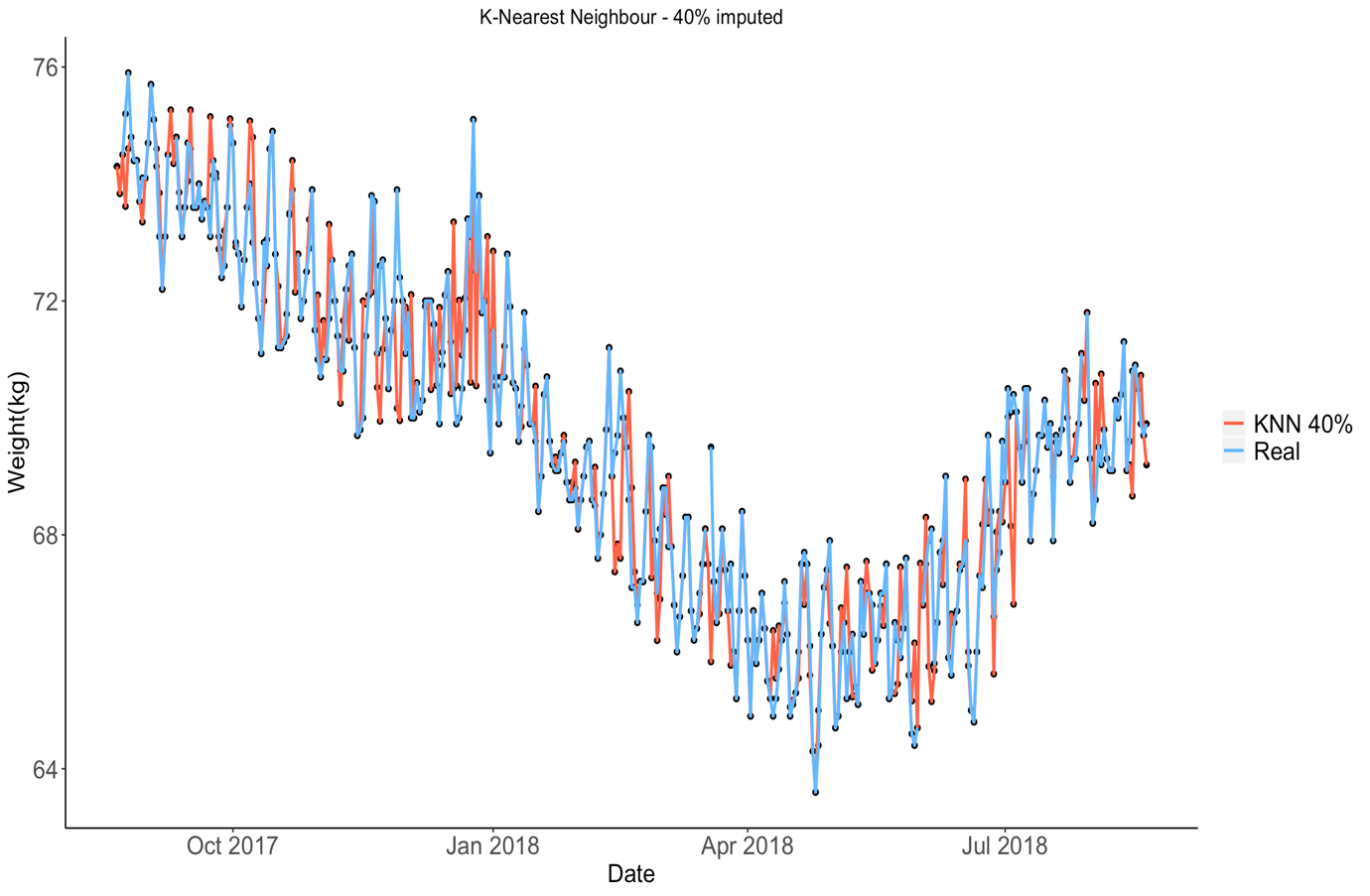


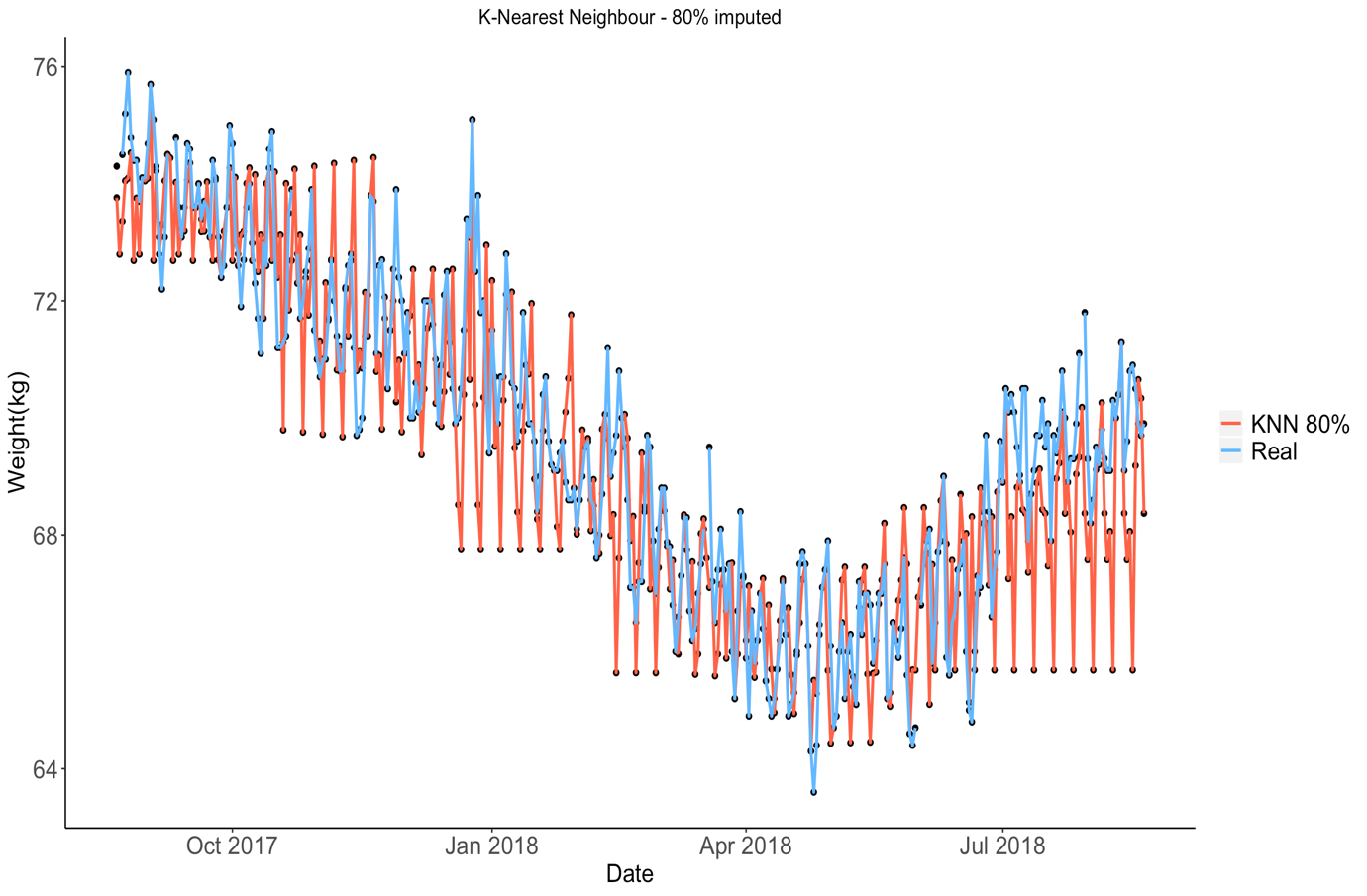


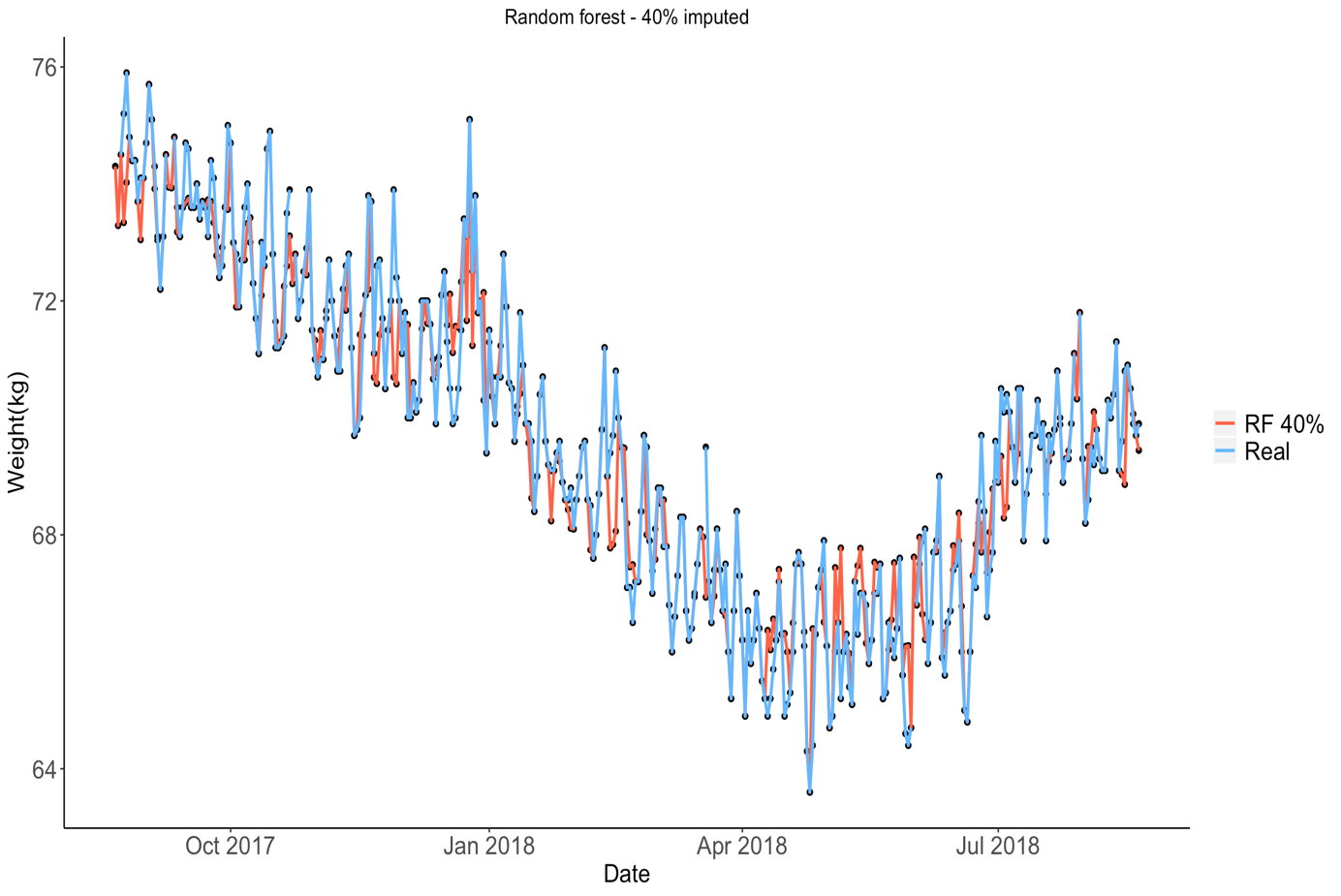


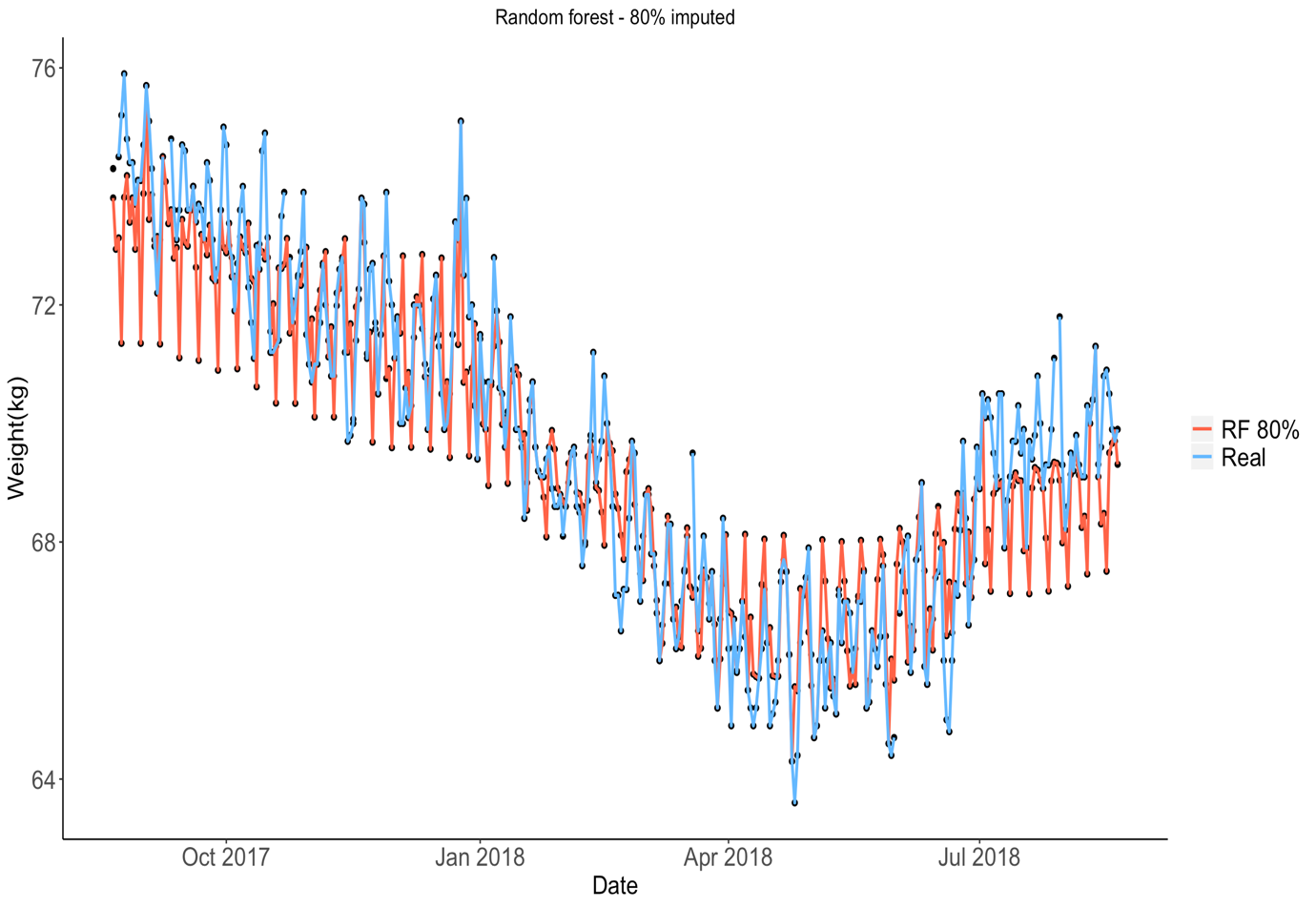

Supplement: Multimedia Appendix 3 [file mhealth_v8i9e17977_app3.docx]
