# Supplementary material for: Data Imputation and Body Weight Variability Calculation Using Linear and Nonlinear Methods in Data Collected From Digital Smart Scales: Simulation and Validation Study
Source: JMIR Mhealth Uhealth. 2020 Sep 11;8(9):e17977. doi: 10.2196/17977 (PMC7519428; doi:10.2196/17977)
Supplement: Multimedia Appendix 4 [file mhealth_v8i9e17977_app4.docx]

Data Imputation and Body Weight Variability Calculation Using Linear and Nonlinear Methods in Data Collected From Digital Smart Scales: Simulation and Validation Study

Multimedia Appendix 4

| Imputation performance across varying conditions of missingness and methods of simulation | | | | | | | | | | | |
| --- | --- | --- | --- | --- | --- | --- | --- | --- | --- | --- | --- |
|  |  | Imputation strategy (RMSE (se)) | | | | | | | | | |
| Missing-ness | Simu-lation | ASSRKS | EWMA | Linear Int | Spline Int | Stine Int | SMKS | TS Clean | KNN | PMM | RF |
|  |  |  |  |  |  |  |  |  |  |  |  |
| 20 | RPM | 0.28 (0.01) | 0.24 (0) | 0.24 (0) | 0.31 (0) | 0.24 (0) | 0.24 (0) | 0.25 (0) | 0.36 (0) | 0.41 (0.01) | 0.33 (0) |
| 40 | RPM | 0.61 (0.07) | 0.37 (0) | 0.37 (0) | 0.53 (0.01) | 0.38 (0) | 0.36 (0) | 0.38 (0) | 0.56 (0) | 0.61 (0.01) | 0.49 (0) |
| 60 | RPM | 1.07 (0.13) | 0.48 (0) | 0.48 (0) | 0.73 (0.01) | 0.49 (0) | 0.46 (0) | 0.48 (0) | 0.78 (0.01) | 0.8 (0.01) | 0.63 (0.01) |
| 80 | RPM | 2.08 (0.21) | 0.66 (0.01) | 0.63 (0.01) | 1.13 (0.02) | 0.65 (0.01) | 0.6 (0) | 0.63 (0.01) | 1.1 (0.01) | 1.03 (0.01) | 0.87 (0.01) |
| 20 | MCAR | 0.3 (0.02) | 0.23 (0) | 0.23 (0) | 0.29 (0) | 0.24 (0) | 0.23 (0) | 0.25 (0) | 0.36 (0) | 0.41 (0.01) | 0.33 (0) |
| 40 | MCAR | 0.48 (0.03) | 0.35 (0) | 0.36 (0) | 0.48 (0) | 0.36 (0) | 0.35 (0) | 0.36 (0) | 0.56 (0) | 0.6 (0.01) | 0.48 (0) |
| 60 | MCAR | 0.98 (0.12) | 0.47 (0) | 0.47 (0) | 0.7 (0.01) | 0.48 (0) | 0.45 (0) | 0.47 (0) | 0.77 (0.01) | 0.78 (0.01) | 0.63 (0.01) |
| 80 | MCAR | 2.55 (0.26) | 0.64 (0.01) | 0.62 (0.01) | 1.07 (0.02) | 0.64 (0.01) | 0.59 (0) | 0.62 (0.01) | 1.07 (0.01) | 1.01 (0.01) | 0.85 (0.01) |

Results of imputation of body weight data from 8,000 simulated data sets generating using multiple methods of simulation and various degrees on missingness. Performance is reported as root mean square error (standard error). Ten imputation strategies are reported. Abbreviations: RPM (real patterns of missingness); MCAR (missing completely at random); Int (interpolation); ASSRKS (ARIMA state-space representation and Kalman smoothing); EWMA (Exponentially weighted moving average); KNN (K-Nearest neighbours); PMM (Predictive means matching); RF (random forest); SMKS (Structural modelling with Kalman smoothing); RMSE (Root mean square error)
