# Supplementary material for: Data Imputation and Body Weight Variability Calculation Using Linear and Nonlinear Methods in Data Collected From Digital Smart Scales: Simulation and Validation Study
Source: JMIR Mhealth Uhealth. 2020 Sep 11;8(9):e17977. doi: 10.2196/17977 (PMC7519428; doi:10.2196/17977)

Data Imputation and Body Weight Variability Calculation Using Linear and Nonlinear Methods in Data Collected From Digital Smart Scales: Simulation and Validation Study

Multimedia Appendix 5

Mean absolute percentage errors associated with the estimating of WV following imputation.


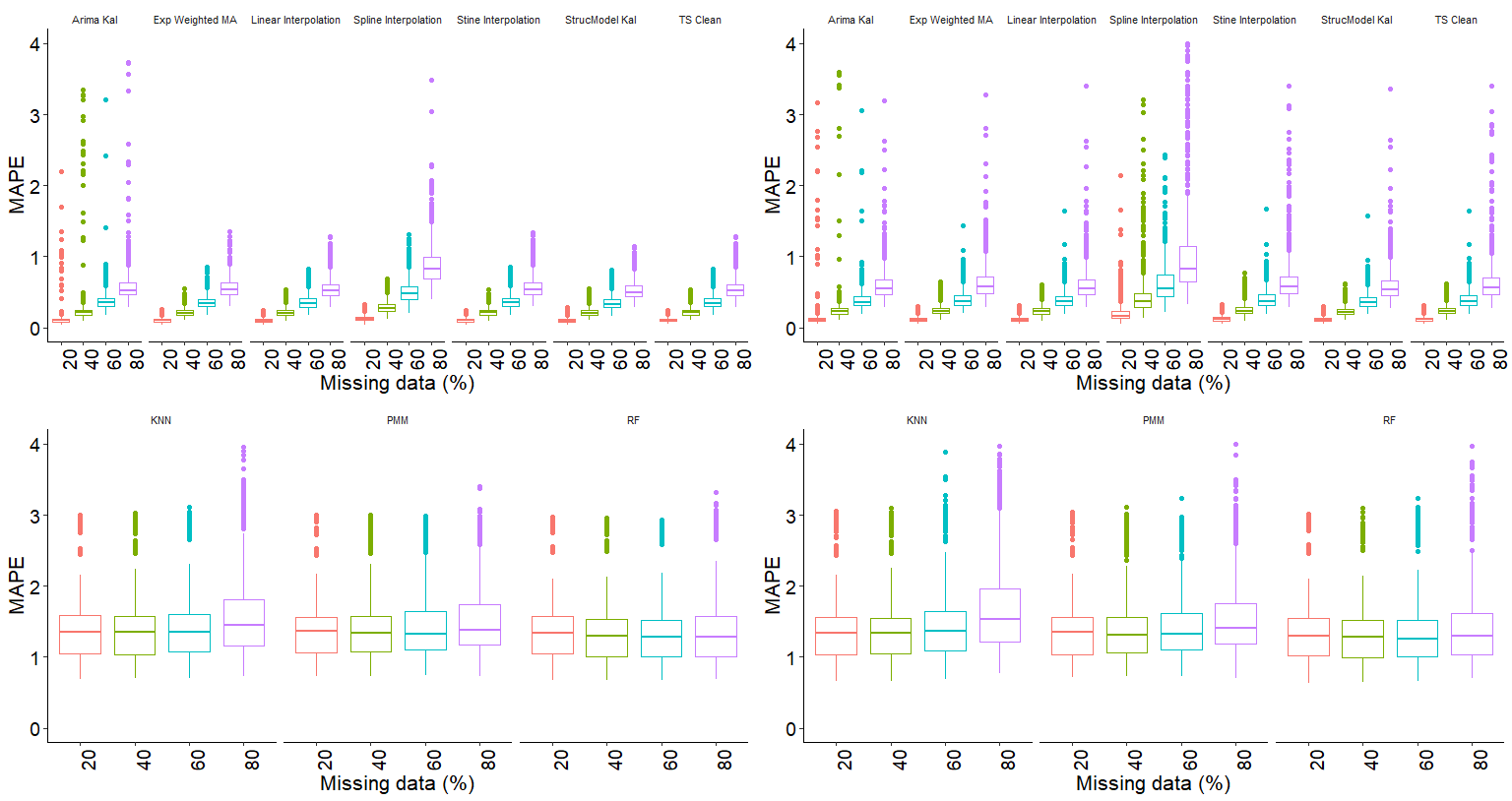


Mean absolute errors associated with the estimating of WV following imputation.


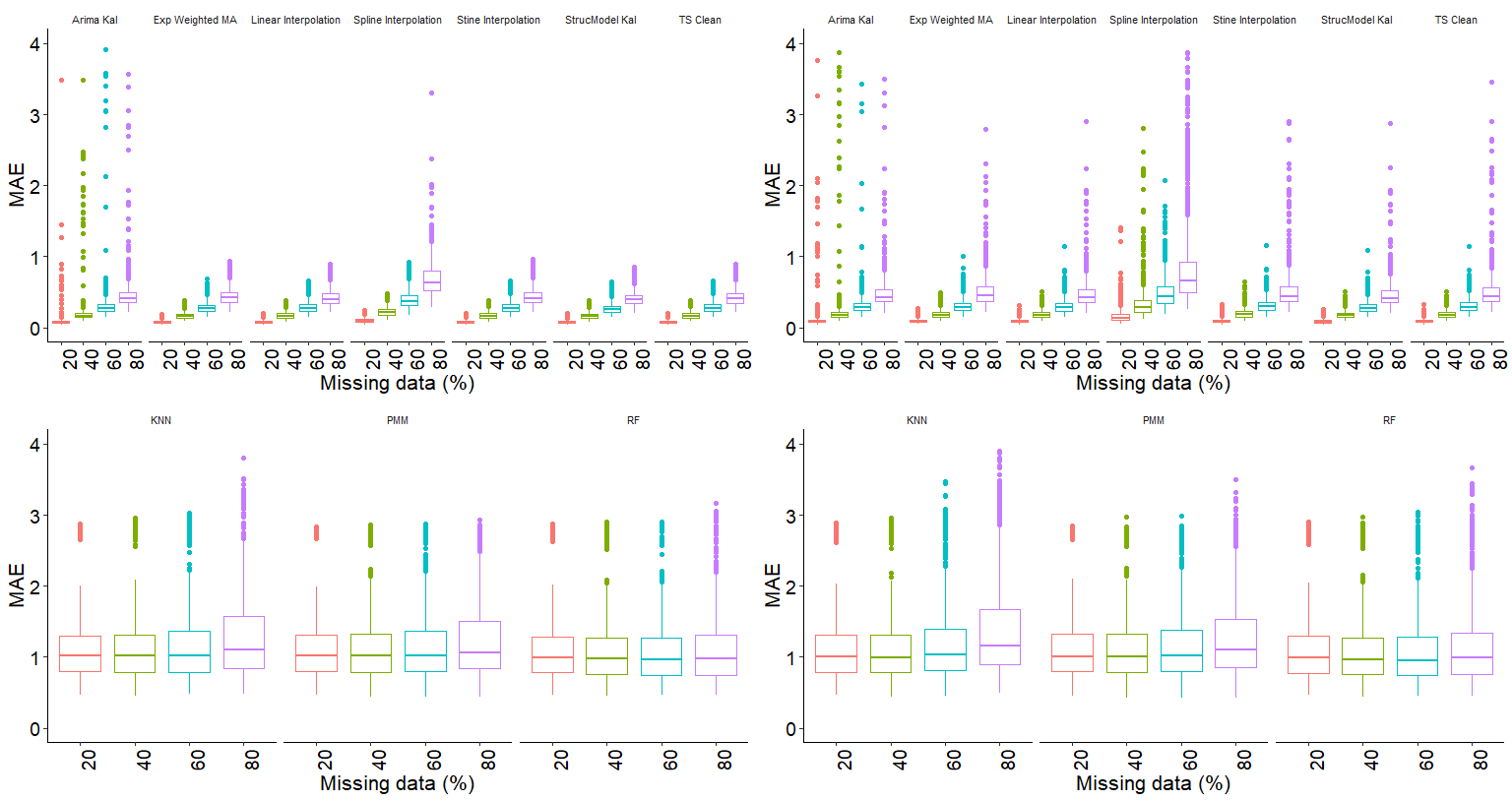

Supplement: Multimedia Appendix 5 [file mhealth_v8i9e17977_app5.docx]
