# Supplementary material for: Data Imputation and Body Weight Variability Calculation Using Linear and Nonlinear Methods in Data Collected From Digital Smart Scales: Simulation and Validation Study
Source: JMIR Mhealth Uhealth. 2020 Sep 11;8(9):e17977. doi: 10.2196/17977 (PMC7519428; doi:10.2196/17977)
Supplement: Multimedia Appendix 6 [file mhealth_v8i9e17977_app6.docx]

Data Imputation and Body Weight Variability Calculation Using Linear and Nonlinear Methods in Data Collected From Digital Smart Scales: Simulation and Validation Study

Multimedia Appendix 6

| Impact of missing data and data imputation on estimation of body weight variability | | | | | | | | | | | | |
| --- | --- | --- | --- | --- | --- | --- | --- | --- | --- | --- | --- | --- |
| Imputation method (mean % error (se)) | | | | | | | | | | | | |
| WV | Missing-ness | None | ASSRKS | EWMA | Linear Int | Spline Int | Stine Int | SMKS | TS Clean | KNN | RF | PMM |
|  |  |  |  |  |  |  |  |  |  |  |  |  |
| NLMD | 20 | -0.4 (0.1) | -5.5 (0.3) | -7.3 (0.1) | -4.1 (0.1) | 6.4 (0.2) | -2.8 (0.1) | -9.3 (0.1) | -4.8 (0.1) | -6.9 (0.1) | -12.3 (0.2) | -7.5 (0.2) |
| RMSE | 20 | -0.2 (0.1) | -0.5 (0.3) | -2.3 (0.1) | -1.1 (0.1) | 4.8 (0.2) | -0.6 (0.1) | -3 (0.1) | -2 (0.1) | -3.4 (0.1) | -6.9 (0.1) | -8.2 (0.1) |
| NLMD | 40 | -1.3 (0.1) | -11.2 (0.4) | -12.9 (0.1) | -8.7 (0.1) | 8.9 (0.2) | -6.2 (0.1) | -20.3 (0.3) | -9.7 (0.1) | -10.2 (0.2) | -24.8 (0.3) | -14.4 (0.4) |
| RMSE | 40 | -0.4 (0.1) | -2.2 (0.4) | -4.3 (0.1) | -2.8 (0.1) | 6.4 (0.2) | -1.9 (0.1) | -6.6 (0.2) | -3.9 (0.1) | -6.6 (0.1) | -13.7 (0.2) | -16.8 (0.2) |
| NLMD | 60 | -2.3 (0.1) | -16.6 (0.6) | -16.7 (0.2) | -14.6 (0.2) | 13.2 (0.2) | -10.2 (0.2) | -37.7 (0.5) | -16 (0.2) | -8.2 (0.3) | -39.1 (0.5) | -22.1 (0.6) |
| RMSE | 60 | -0.6 (0.1) | -3.2 (0.5) | -5.5 (0.2) | -4.7 (0.1) | 9.1 (0.2) | -3.3 (0.1) | -11.4 (0.3) | -6 (0.2) | -10.2 (0.2) | -22.7 (0.3) | -29.3 (0.4) |
| NLMD | 80 | -7 (0.2) | -27.1 (1) | -18.7 (0.3) | -30.3 (0.4) | 20.3 (0.4) | -20.6 (0.3) | -84.9 (1.4) | -34.4 (0.5) | 1.9 (0.5) | -49.4 (0.8) | -29.5 (0.8) |
| RMSE | 80 | -3.2 (0.2) | -3.5 (0.7) | -6.3 (0.2) | -9.6 (0.3) | 15.2 (0.4) | -6.7 (0.2) | -23.1 (0.6) | -11.6 (0.3) | -16.9 (0.4) | -38.7 (0.5) | -51.7 (0.6) |

Mean deviation (%) between true weight variability estimates from observed data and that estimated from simulated and imputed data sets. Performance is reported as root mean square error (standard error). Ten imputation strategies are reported. Abbreviations: NLMD (non-linear mean deviation); RMSE (root mean square error); Int (interpolation); ASSRKS (ARIMA state-space representation and Kalman smoothing); EWMA (Exponentially weighted moving average); KNN (K-Nearest neighbours); PMM (Predictive means matching); RF (random forest); SMKS (Structural modelling with Kalman smoothing); RMSE (Root mean square error).
